# Analysis of an inventory system with emergency ordering option at the time of supply disruption

**DOI:** 10.1007/s00291-021-00636-x

**Published:** 2021-06-04

**Authors:** Saeed Poormoaied, Ece Zeliha Demirci

**Affiliations:** 1 Industrial Engineering and Innovation Sciences, Eindhoven University of Technology, Eindhoven, The Netherlands; 2 Department of Industrial Engineering, TED University, Ankara, Turkey

**Keywords:** Inventory, Supply disruption, Continuous-review inventory policy, CTMC

## Abstract

This paper studies a continuous-review stochastic inventory problem for a firm facing random demand and random supply disruptions. The supplier experiences operational (*on*) and disrupted (*off*) periods with exponentially distributed durations. The firm adopts an order-up-to level policy during the *on* period and additionally can release an emergency order based on the inventory level just before disruption. This inventory policy is described by a continuous-time Markov chain model. We analyze the model for two different lead time scenarios and suggest solution approaches yielding the optimal policy parameters. In a numerical study, we explore the value of exercising such a policy and show that an emergency ordering opportunity at the disruption time brings substantial cost savings in cases with high lost sales cost, long *off* period, and low percentage of supplier’s availability.

## Introduction

Supply chains need to consider supply uncertainty during their planning phase to avoid the potential risks of incurring high operating costs and having low customer service level. Supply uncertainty has been categorized into different forms in the literature such as yield, capacity, lead time, input cost, and supply disruption uncertainties (Snyder et al. 2016). The supply disruptions are discrete and random events that make the supplier stop functioning completely or partially for a random amount of time.

The reason behind supply disruptions can be classified under two groups (Atasoy et al. 2012): (1) unpredictable disruptions due to natural disasters, terrorist attacks, labor actions, transportation disruptions, and (2) predictable disruptions that arise due to capacity restrictions, scarcity of some resources and supplier’s capacity allocation scheme.

Although supply disruptions are rare, their consequences can be massive if companies do not have effective mitigation strategies. Consider the disruption in Philips Electronics in the year 2000 which was due to a fire at the company’s facility in New Mexico (Xia et al. 2004). At that time, the productions of Nokia and Ericson’s mobile phones were dependent on the supply of computer chips by Philips. Philips was unable to supply the chips for several weeks. Different mitigation strategies implemented by the two companies have led to completely different results. While Ericson’s mobile phone division lost 1.68 billion dollars, Nokia increased its global market share by 3%. Toyota Motor also reported the after-effects of the earthquake and tsunami that hit Japan in 2011 (Forbes 2011). The company lost two billion dollars in revenue due to experiencing severe shortages in materials around the world. Moreover, Hendricks and Singhal (2005) have empirically shown that a firm suffering from disruption could encounter 33–40% decrease in stock returns in the long run. A recent example is the Covid-19 pandemic, which has severely affected production and distribution activities in both domestic and global supply chains. A survey conducted by Institute for Supply Chain Management (ISM) over 600 US companies revealed that the majority of suppliers are operating on average with 50% capacity, which resulted in longer final product lead times and negative revenue impact of 5.6–15% (ISM 2020). Similarly, around 35% of manufacturers in India reported disturbances in their manufacturing processes (Agrawal et al. 2020).

Snyder et al. (2016), Tomlin (2006), and Kleindorfer and Saad (2005) have discussed several strategies against supply disruptions, two of which are: overstocking and information sharing. These two strategic remedies are considered throughout our paper. In practice, buyers are unaware of disruptions until their orders are not satisfied, whereas suppliers generally have information about the likelihood and timing of disruptions. Suppliers are prone to disruptions due to scarcity of some resources (such as shortage in raw materials), capacity restrictions, planned maintenance, labor strike, etc. Although suppliers have prior information on such disruptions with certainty, the time between two consecutive disruptions is random and the exact time can only be noticed just before it occurs. As the disruption information is significantly valuable for the buyers, they have started to monitor their suppliers for warnings of potential supply discontinuity problems (Tomlin and Snyder 2006). For instance, Open Ratings have developed a supply chain monitoring software that will enable firms to monitor their suppliers. The software allows firms to anticipate potential disruptions and take actions accordingly. Therefore, we assume that the supplier can predict the disruptions and the buyer gets informed when disruption occurs or is about to occur.

In this paper, we suggest an inventory policy that can be exercised by a buyer who experiences supply disruptions. The policy provides the buyer an emergency order opportunity right before or just when the supplier gets disrupted. Requesting emergency orders at the beginning of the disruption period can avoid unduly shortages when the retailer’s stock level is low and there is a potential for a long disruption period. The suggested policy is consistent with the proactive behaviors against supply disruptions that are observed in practice. Examples include inventory hoarding at the beginning of an epidemic like Covid-19 (Ivanov and Das 2020) and defensive purchases of wholesalers right after the flood ravaged Ayutthaya, Thailand in 2011 (NATIONS U, 2013). The emergency ordering option incurs additional costs compared to regular orders as the supplier is in a special mode. Therefore, the buyer needs to make a trade-off between lower stock-out probabilities and the extra cost of placing an emergency order (Poormoaied 2020; Poormoaied et al. 2020b). We aim to address the settings under which emergency order opportunity is valuable and also quantify its benefits.

To perform our analysis, we consider a setting consisting of a retailer facing both random demand and random supply disruption. We explore the problem for a single-item and continuous-review inventory system. The supply process alternates among two states that are the *on* period, during which it functions normally, and the *off* period, during which it is disrupted. We assume that the durations of *on* and *off* periods are exponentially distributed, and the demand follows the Poisson distribution. Similar to the paper of Parlar and Berkin (1991), the decision-maker is aware of the availability status of the supplier at any time point. We suggest an inventory policy, which uses an order-up-to level policy during the *on* period and at the beginning of the *off* period, but with different policy parameters. The policy parameters of the *on* period are denoted by $\,\,\,\,\,\,\,(s_1,S_1)\,\,\,\,\,$, whereas the ones at the beginning of *off* period are shown by $(s_2,S_2)$. We refer to this policy as $(s_1,S_1,s_2,S_2)$ policy and it works as follows. If the inventory level is at or below $s_1$ and the supply is available, place an order to bring the inventory level up to $S_1$. If the inventory level is at or below $s_2$ at the beginning of the disruption period, place an order to bring the inventory level up to $S_2$. We assume that the supplier has ample capacity and all replenishments are complete. In addition, any unmet demand is lost and the supplier incurs goodwill loss cost for each unit of unmet demand. The cost factors taken into account are fixed and variable costs of regular and emergency orders, inventory holding cost, and lost sales cost. Throughout the paper, we consider emergency lead time as zero and examine analytically two different scenarios of regular lead times, which are (1) zero and (2) exponentially distributed. We further simulate the case with a positive constant regular lead time. We follow the same procedure for analytical analyses of cases (1) and (2). We first build the CTMC model of $(s_1,S_1,s_2,S_2)$ policy and then develop an algorithm to find steady-state probabilities of the system. The algorithm yields the stationary distribution of the inventory level and the resulting distribution is used to derive operating characteristics and the expected total operating cost of the inventory system. As it is quite challenging to derive explicit expressions for the optimal policy parameters, we develop an enumeration-based algorithm to calculate them.

We conduct a numerical analysis to illustrate the value of disruption risk information on the performance of the proposed inventory policy. In general, the results show similar insights for all lead time scenarios. Our findings demonstrate that the emergency ordering option is not used unless the lost sales cost is sufficiently high. It is especially efficient when there is a high chance of being disrupted for a long period. In those cases, emergency ordering cost is justified by the reduction in lost sales cost.

We contribute to the literature of inventory models under the threat of disruptions in two dimensions: (1) We expand the analytical model of the continuous review order-up-to level inventory system with random demand and supply to a broader extent by considering zero and exponentially distributed lead times. (2) The supply chain disruption literature has explored the value of disruption information only for deterministic demand setting (Bakal et al. 2017). However ,in this study, we conduct an analysis with stochastic demand. In addition to proposing a new inventory policy to mitigate the supply discontinuity risk, we also assess the value of sharing disruption information with the buyer for a continuous-review inventory system.

The remainder of the paper is organized as follows. We begin the paper in Sect. 2 by providing the literature review on inventory models subject to the threat of disruption. We develop a CTMC model of the inventory policy in Sect. 3 and derive the steady-state probabilities. We also present an enumeration-based solution approach to find the inventory policy parameters. In Sect. 4, we extend the CTMC model for the exponential regular lead time case. In Sect. 5, we conduct the numerical study that enriches our understanding of the suggested inventory policy and its financial impact. Finally, we provide concluding remarks and directions for future research in Sect. 6. We relegate all proofs to "Appendix 5."

## Literature review

Supply chain disruptions have inspired an increasing number of papers since 1998. A detailed review of studies in this field can be found in Snyder et al. (2016), which is organized according to the categories of mitigation strategies developed against supply chain disruptions. Our study falls into the category of mitigating disruptions through inventories. Thus, we limit our discussion on the inventory models under the threat of disruptions. These inventory models can be categorized with respect to several dimensions as within classical inventory models, which are mainly periodic vs. continuous-review, backorders vs. lost sales, single vs. multi-echelon, cost structures, and decision variables.

The basic continuous-inventory model is the classical economic order quantity (EOQ) model. The study of Parlar and Berkin (1991) is the first to introduce disruptions into this model, which has been referred to as EOQ with Disruptions (EOQD) in the literature. They consider an EOQ setting with lost sales and assume that *on* and *off* periods have random lengths. They utilize the renewal reward theorem to find the expression for the expected cost per unit time. However, Berk and Arreola-Risa (1994) show that their model is incorrect due to implicit assumptions on stock-outs and usage of shortage cost. They present the corrected model by assuming exponentially distributed *on* and *off* periods. Their new cost function is quasi-convex and thus an exact expression for the optimal policy parameter cannot be derived. Later, Snyder (2014) presents a simple but effective approximation to find the optimal order quantity for the EOQD model formulated in Berk and Arreola-Risa (1994). Recently, Bakal et al. (2017) consider EOQD with an additional order opportunity for the buyer when the supplier gets disrupted. They explore the benefits of an additional order opportunity as well as the value of disruption information. Under the classical EOQ model, a zero inventory ordering policy (i.e., an order is placed when the inventory level hits zero) is optimal. However, it may be preferred to have a positive inventory level at the disruption point under EOQD. Parlar and Perry (1995), Parlar and Perry (1996), and Heimann and Waage (2007) study an extension of EOQD model with nonnegative reorder point. In addition to these studies, many authors have worked on EOQD whose detailed discussion is found in Snyder et al. (2016).

A large percentage of the existing inventory models subject to supply disruptions have assumed that a specific inventory control policy is exercised by the buyer when the supplier is available. The policies considered are (*s*, *S*) policy (e.g., Kalpakam and Sapna (1997), Moinzadeh and Aggarwal (1997), and Arreola-Risa and DeCroix (1998)), (*s*, *Q*) policy (e.g., Gupta (1996), Parlar (1997), Mohebbi (2004), Mohebbi and Hao (2006), and Mohebbi and Hao (2008)) or the base-stock policy (e.g., Atan and Rousseau (2016) and Atan and Snyder (2012)). Different from an ordinary inventory model in which the supplier is always available, they consider the case where the supplier might be unavailable and does not satisfy any demand during its *off* period. The primary focus of this line of studies is to find optimal parameters of the assumed policy by incorporating supply interruptions into the inventory model. The main differences among these studies are in terms of system characteristics considered such as demand distribution, lead times, and alternating processes that characterize supplier’s *on* and *off* periods. Their main conclusion is that ignorance of supply disruptions while determining the policy parameters results in higher operating costs, especially when the stock-out cost is high and disruption periods are long.

The study of Kalpakam and Sapna (1997) is among the earliest papers addressing the supply disruption problem within the context of continuous review order-up-to level inventory policies. They consider a setting with lost sales, zero lead time, unit renewal demands, and exponentially distributed *on* and *off* periods. They develop an efficient algorithm to find the reorder and order-up-to levels. Moinzadeh and Aggarwal (1997) study an unreliable production/inventory system with constant demand and production rates that is subject to interruptions. They assume that the length of the disruption period is exponentially distributed and excess demand is backordered. They propose the employment of an order-up-to level policy, (*s*, *S*), for such settings and derive operating characteristic expressions to evaluate the expected cost rate of the system. Later, Arreola-Risa and DeCroix (1998) also suggest using (*s*, *S*) policy for a system facing Poisson demand and exponentially distributed *on* and *off* periods. Different from previous studies they assume partial backordering and zero replenishment lead time. They find out the optimal policy parameters using the exact cost expression and provide insights into the optimal inventory strategy when there are changes in the parameters of the system.

In light of the previous work within the context of a continuous review order-up-to level inventory system subject to random supply disruptions, our paper makes a number of contributions. We expand the analytical framework of the supply-interruption problem to a broader extent by considering a setting with random demand and zero, positive constant, and exponentially distributed lead times. We derive exact cost expressions for zero and exponentially distributed lead time cases and also develop solution approaches to find the optimal policy parameters. In addition, we develop a simulation model for the case with fixed lead time. We propose an alternative inventory policy that can be exercised by a buyer in such a setting. This new policy allows the buyer to exercise two different reorder and order-up-to levels, one during the *on* period and one at the beginning of the *off* period. We refer to the order given at the beginning of the *off* period as the emergency order. Note that emergency shipments provide a powerful mechanism to alleviate the risk of stock-outs and can result in substantial benefits (Poormoaied et al. 2020a). Through extensive numerical experiments, we observe that if the supplier is able to deliver an additional order at the disruption point, the buyer avoids the risk of incurring high lost sales cost. Besides, the buyer does not need to search for an alternative supplier.

## Model description and analysis

In this section, we describe the system characteristics and suggest an inventory control policy to be exercised by a retailer (she) whose supplier (he) is subject to random disruptions. We present a CTMC model to analyze how the inventory policy operates and provide an algorithm to calculate the steady-state probabilities. The resulting distribution is used to derive exact functional forms of the operating characteristics and hence the expected total cost. Last, we present an optimization problem to find the optimal policy parameters.

### Inventory policy

We study a single-location single-item inventory system that includes a retailer (buyer) who faces stochastic demand and a supplier who is subject to random interruptions. Table 1 presents the notations and definitions used in developing the model. Demand at the retailer follows a Poisson process with rate $\lambda$. The supplier goes through available (*on*) and unavailable (*off*) periods. The lengths of *on* and *off* periods are independent and follow exponential distribution with rates $\mu$ and $\nu$, respectively. Once the supplier switches to the *off* mode, no order is accepted. The supplier becomes available as soon as he switches to the *on* mode again.

We suggest an inventory policy for the retailer, which provides an opportunity to place an emergency order right before or just as the supplier gets disrupted. Thus, the imminent shortages during the *off* period are prevented. We call this policy $(s_1,S_1,s_2,S_2)$ policy, where $s_1$ and $s_2$ are reorder levels, and $S_1$ and $S_2$ are order-up-to levels of *on* and *off* periods, respectively. The policy can be summarized as follows: (1) when the inventory level is at or below $s_1$ and the supplier is *on*, a regular order is placed to increase the inventory level up to $S_1$; (2) when the supplier’s state turns into *off* and at that point the inventory level is at or below $s_2$, an emergency order is requested to raise the inventory level up to $S_2$. The proposed policy reduces to the classical order-up-to level policy of (*s*, *S*) if the chance of having an *off* period is very small.

**Table 1:** Notation

$\lambda$	Poisson demand rate of the retailer.
$\mu$	Exponential rate of *on* period length, expected length $=\mu ^{-1}$.
$\nu$	Exponential rate of *off* period length, expected length $=\nu ^{-1}$.
$S_1$	Order-up-to level for regular replenishment.
$s_1$	Reorder level for regular replenishment.
$S_2$	Order-up-to level for emergency replenishment.
$s_2$	Reorder level for emergency replenishment.
$L_o$	Regular replenishment lead time.
*b*	Unit lost sales cost of the buyer.
*h*	Unit holding cost of the buyer.
$K_o$	Fixed cost of regular ordering.
$K_e$	Fixed cost of emergency ordering.
$c_o$	Unit purchasing cost of a regular order.
$c_e$	Unit purchasing cost of an emergency order.
$\mathcal {P}_{(i,k)}$	The fraction of time in the long run that the inventory system is in state (*i*, *k*),
	$i=\{0,1,2,\ldots ,\max (S_1,S_2)\}$ and $k=\{F,N\}$
	(*F* denotes *off* mode and *N* denotes *on* mode).
$\pi _i$	Steady-state probability that the inventory level of the retailer is *i*,
	$\pi _i=\mathcal {P}_{(i,N)}+\mathcal {P}_{(i,F)}$, $i=\{0,1,2,\ldots ,\max (S_1,S_2)\}$.
$\mathbb {E}[RO]$	Expected regular ordering cost.
$\mathbb {E}[EO]$	Expected emergency ordering cost.
$\mathbb {E}[OH]$	Expected on-hand inventory.
$\mathbb {E}[LS]$	Expected number of lost sales.
$\mathbb {E}[C]$	Expected total cost per unit time.

We assume that there can be at most one outstanding order at any time. It is a common assumption among inventory models with disruptions as it allows analytical tractability. Any unsatisfied demand during both *on* and *off* periods is lost. Moreover, the regular and emergency order replenishments are taken as complete. We first develop the model under the assumption of negligible lead time for both regular and emergency shipments. This implies that any order is delivered instantaneously upon its acceptance given that the supplier is in the *on* period or at the beginning of the *off* period. We then extend our model to the exponentially distributed regular lead time case in Sect. 4. Moreover, the setting with positive constant regular lead time is simulated in Sect. 5.2. It is assumed that the emergency lead time is zero throughout the paper. This assumption is reasonable as the supplier expects to receive an emergency order of size less than $S_2$ at the beginning of the *off* period. He can process these orders either by reserving on-hand inventory of at most $S_2$ or by being prepared based on prior information on disruption. Thus, he asks a higher fixed ordering cost for emergency orders compared to regular ones.

The assumptions of having at most one outstanding order and lost sales imply that $s_1 < S_1$. Also, we have $s_2 \le S_2$ as a natural assumption of order-up-to policies. Since the replenishments are instantaneous and regular replenishments are less costly, the buyer would always prefer to give a regular order instead of an emergency order if possible. This implies that the relation $s_1\le s_2$ needs to hold. When $s_1=s_2$, the buyer would choose to give regular order just before the disruption. Thus, in this case the emergency ordering opportunity would not be utilized. We did not impose any relation between emergency and regular order-up-to levels, i.e., either of the relations $S_2 \le S_1$ or $S_2>S_1$ holds. Thus, the maximum inventory level at any time point is $\max (S_1,S_2)$. Moreover, when $s_2=S_2$, the $(s_1,S_1,s_2,S_2)$ policy reduces to the (*s*, *S*) policy.

**Fig. 1:**
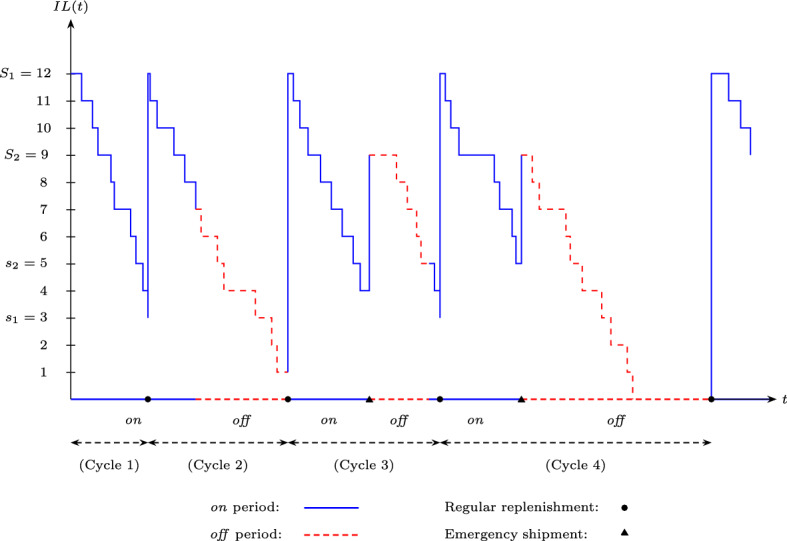
A sample path of the inventory system operating under the $(s_1,S_1,s_2,S_2)$ policy

Figure 1 illustrates a typical realization of the inventory process and shows how the retailer’s ordering policy works. Here, we denote the inventory level at time *t* by *IL*(*t*) and take $s_1=3$, $S_1=12$, $s_2=5$, and $S_2=9$. We define a *renewal cycle* as the time between two successive regular orders, i.e., the time between two bullet points ($\bullet$) in Fig. 1. In other words, starting at an inventory level of $S_1$, a renewal cycle is the time required for the inventory level to be equal to $S_1$ again. Typical behaviors during cycles can be described as follows. If the supplier is *on* and the inventory level hits $s_1$, a regular order is placed to raise the inventory level to $S_1$ (see Cycle 1). At the time point supplier becomes *off*, (1) if the inventory level is at or below $s_2$, place an emergency order to bring the inventory level up to $S_2$ (see Cycle 3), and (2) do not order otherwise (see Cycle 2). When the supplier’s state turns to *on*; (1) no regular order is placed if the inventory level is above $s_1$ (see Cycle 3), (2) a regular order is placed to increase the inventory level up to $S_1$ if the inventory level is at or below $s_1$ (see Cycles 2 and 4). A stock-out situation is observed within Cycle 4, during which any demand is lost.

The cost components considered are as follows: penalty cost per unit of unmet demand (*b*), holding cost per each unit of inventory held per unit time (*h*), fixed costs of placing a regular order and an emergency order ($K_o$ and $K_e$, respectively), and unit purchasing costs for a regular order and an emergency order ($c_o$ and $c_e$, respectively).

### CTMC model of $(s_1,S_1,s_2,S_2)$ policy with zero lead time

We model the inventory policy under consideration as CTMC, where the state of the system is described by the inventory level and the mode of the supplier. Specifically, the state of the system is defined by $\{\mathcal {A}(t),\mathcal {B}(t);\, t \ge 0\}$, where $\mathcal {A}(t)=\{0,1,2,\ldots ,\max (S_1,S_2)\}$ and $\mathcal {B}(t)=\{F,N\}$ represent the inventory level and the mode of the supplier at time *t*, respectively (*F* denotes *off* period and *N* corresponds to *on* period). Note that we exclude states (*i*, *N*) for $i=0,1,\ldots ,s_1$ as these states are not observed by the system. Therefore, we have $M=s_1+1+2(\max (S_1,S_2)-s_1)$ states in total.

Table 2 represents the transition rates among states of the system. There are seven types of transitions in total that can be detailed as follows. (1) Type 1: If the inventory level is below $s_1$ while the supplier’s mode switches from *off* to *on*, a regular order is placed immediately to increase the inventory level to $S_1$; (2) Types 2 and 3: A demand arrives with rate $\lambda$ independent of supplier’s state and decreases the inventory level by one; (3) Type 4: If the inventory level is above $s_1$, the supplier switches from *off* to *on* with rate $\nu$ while the inventory level stays the same; (4) Type 5: If the inventory level is above $s_2$, the supplier switches from *on* to *off* with rate $\mu$ while the inventory level stays the same; (5) Type 6: If the inventory level is between $s_1+1$ and $s_2$, the supplier transitions from *on* to *off* with rate $\mu$. In this case, the inventory level increases to $S_2$ with an emergency order; and (6) Type 7: If the inventory level is $s_1+1$ and the supplier is *on*. When a demand arrives (with rate $\lambda$), the inventory level drops to reorder level $s_1$ and hence a regular order is immediately placed to bring the inventory level to $S_1$. You can see "Appendix 1" for the transition diagram of the CTMC under consideration.

**Table 2:** Transition rates of CTMC for zero lead time

Type	From	To	Rate	Range
1	(*i*, *F*)	$(S_1,N)$	$\nu$	$i=0,1,\ldots ,s_1$
2	(*i*, *F*)	$(i-1,F)$	$\lambda$	$i=1,2,\ldots ,S_1$
3	(*i*, *N*)	$(i-1,N)$	$\lambda$	$i=s_1+2,\ldots ,S_1$
4	(*i*, *F*)	(*i*, *N*)	$\nu$	$i=s_1+1,\ldots ,S_1$
5	(*i*, *N*)	(*i*, *F*)	$\mu$	$i=s_2+1,\ldots ,S_1$
6	(*i*, *N*)	$(S_2,F)$	$\mu$	$i=s_1+1,\ldots ,s_2$
7	$(s_1+1,N)$	$(S_1,N)$	$\lambda$	–

Recall that we do not impose any relation between order-up-to levels $S_1$ and $S_2$. In this section, we show how to derive the limiting distribution of inventory level and expected total cost only for $S_2 \le S_1$ case. They are not reported for $S_2 > S_1$ case due to space limitations; however, one can obtain those by following a similar methodology. Note that the transition rates reported in Table 2 hold for both cases.

Let $\mathcal {P}_{(i,k)} \equiv \lim _{t \rightarrow \infty } \mathbb {P}\{\mathcal {A}(t)=i, \mathcal {B}(t)=k\}$ for $i=\{0,1,2,\ldots ,S_1\}$ and $k=\{F,N\}$, be the steady-state probabilities (i.e., the fraction of time in the long run that the system is in state (*i*, *k*)) and $\varvec{\mathcal {P}} = (\mathcal {P}_{(i,F)},\mathcal {P}_{(j,N)})$, for $i=0,1,\ldots ,S_1$ and $j=s_1+1,\ldots ,S_1$ denote the steady-state probability vector. Note that $\varvec{\mathcal {P}}$ is an *M*-dimensional vector and $\mathcal {P}_{(i,N)}=0$ for $i=0,\ldots ,s_1$. The steady-state probabilities are obtained by solving the balance equations. These equations are constructed as follows:

1 $$\begin{aligned} \varvec{\mathcal {P}} \times \varvec{\Lambda } = \varvec{0}, \ \ \varvec{\mathcal {P}} \times \varvec{1} = 1, \end{aligned}$$

where $\varvec{\Lambda }$ is a $M \times M$ matrix standing for the transitions among states, $\varvec{0}$ is the *M*-dimensional row vector $(0,\ldots ,0)$, and $\varvec{1}$ is the *M*-dimensional column vector $(1,\ldots ,1)^T$. The relationships given above translate into the system of linear equations given in (2). These equations ensure that the total rate at which the process enters a specific state is equal to the total rate at which the process leaves that state.

2 $$\begin{aligned} \begin{aligned}&(1) \ \nu \mathcal {P}_{(0,F)} = \lambda \mathcal {P}_{(1,F)}. \\ {}&(2) \ (\nu +\lambda ) \mathcal {P}_{(i,F)} = \lambda \mathcal {P}_{(i+1,F)}, \ \forall \ i=1,\ldots ,s_2. \\ {}&(3) \ (\nu +\lambda ) \mathcal {P}_{(i,F)} = \lambda \mathcal {P}_{(i+1,F)} + \mu \mathcal {P}_{(i,N)}, \ \forall \ i=s_2+1,\ldots ,S_2-1,S_2+1,\ldots ,S_1-1. \\ {}&(4) \ (\nu +\lambda ) \mathcal {P}_{(S_2,F)} = \lambda \mathcal {P}_{(S_2+1,F)} + \mu \Big (\sum _{i=s_1+1}^{s_2}\mathcal {P}_{(i,N)}+\mathcal {P}_{(S_2,N)}\Big ). \\ {}&(5) \ (\nu +\lambda ) \mathcal {P}_{(S_1,F)} = \mu \mathcal {P}_{(S_1,N)}. \\ {}&(6) \ (\mu +\lambda ) \mathcal {P}_{(i,N)} = \lambda \mathcal {P}_{(i+1,N)} + \nu \mathcal {P}_{(i,F)}, \ \forall \ i=s_1+1,\ldots ,S_1-1. \\ {}&(7) \ (\mu +\lambda ) \mathcal {P}_{(S_1,N)} = \lambda \mathcal {P}_{(s_1+1,N)} + \nu \Big (\sum _{i=0}^{s_1}\mathcal {P}_{(i,F)}+\mathcal {P}_{(S_1,F)}\Big ). \end{aligned} \end{aligned}$$

In the following theorem, we show that the Markov chain $\{\mathcal {A}(t),\mathcal {B}(t);\, t \ge 0\}$ is ergodic, which guarantees the existence of the steady-state probabilities.

#### Theorem 1

The Markov chain $\{\mathcal {A}(t), \mathcal {B}(t);\, t \ge 0\}$ is ergodic.

The set of equations given in (2) can be rewritten as follows:

3 $$\begin{aligned} \mathcal {P}_{(i,F)}= & {} \frac{\nu }{\lambda } \Big (\frac{\nu +\lambda }{\lambda }\Big )^{i-1} \mathcal {P}_{(0,F)}, \ \forall \ i=1,\ldots ,s_2. \end{aligned}$$

4 $$\begin{aligned} \mathcal {P}_{(i,F)}= & {} \frac{\lambda }{\nu +\lambda } \mathcal {P}_{(i+1,F)} + \frac{\mu }{\nu +\lambda } \mathcal {P}_{(i,N)}, \ \forall \ i=s_2+1,\ldots ,S_2-1,S_2+1,\ldots ,S_1-1. \end{aligned}$$

5 $$\begin{aligned} \mathcal {P}_{(S_2,F)}= & {} \frac{\lambda }{\nu +\lambda } \mathcal {P}_{(S_2+1,F)} + \frac{\mu }{\nu +\lambda } \Big (\sum _{i=s_1+1}^{s_2} \mathcal {P}_{(i,N)} + \mathcal {P}_{(S_2,N)} \Big ). \end{aligned}$$

6 $$\begin{aligned} \mathcal {P}_{(S_1,F)}= & {} \frac{\mu }{\nu +\lambda } \mathcal {P}_{(S_1,N)}. \end{aligned}$$

7 $$\begin{aligned} \mathcal {P}_{(i,N)}= & {} \frac{\lambda }{\mu +\lambda } \mathcal {P}_{(i+1,N)} + \frac{\nu }{\mu +\lambda } \mathcal {P}_{(i,F)}, \ \forall \ i=s_1+1,\ldots ,S_1-1. \end{aligned}$$

8 $$\begin{aligned} \mathcal {P}_{(S_1,N)}= & {} \frac{\lambda }{\mu +\lambda } \mathcal {P}_{(s_1+1,N)} + \frac{\nu }{\mu +\lambda } \Big (\sum _{i=0}^{s_1} \mathcal {P}_{(i,F)} + \mathcal {P}_{(S_1,F)} \Big ). \end{aligned}$$

9 $$\begin{aligned} \sum _{i=0}^{S_1} \mathcal {P}_{(i,F)} + \sum _{i=s_1+1}^{S_1} \mathcal {P}_{(i,N)}= & {} 1. \end{aligned}$$

We define $\pi _i$ as the steady-state probability corresponding to inventory level *i* (i.e., the fraction of time in the long run that the inventory level is equal to *i*). It follows that

10 $$\begin{aligned} \begin{aligned}&\pi _i = \mathcal {P}_{(i,F)}, \ \forall \ i=0,1,\ldots ,s_1, \\ {}&\pi _i = \mathcal {P}_{(i,F)} + \mathcal {P}_{(i,N)}, \ \forall \ i=s_1+1,\ldots ,S_1, \end{aligned} \end{aligned}$$

where $$\sum _{i=0}^{S_1} \pi _i =1$$.

It is not possible to express steady-state probabilities with explicit expressions; instead, we develop recursive equations that enables us to determine them in four steps. For ease of exposition, we use the following notation:

$$\begin{aligned} c_1 = \frac{\mu }{\nu +\lambda }, \ c_2 = \frac{\lambda }{\mu +\lambda }, \ c_3 = \frac{\lambda }{\nu +\lambda }, \ \text {and} \ c_4 = \frac{\nu }{\mu +\lambda }. \end{aligned}$$

*Algorithm for determining steady-state probabilities: *

*Step 1.* Considering the sets of equations given in (4) and (7) together, for $i=S_2+1,\ldots ,S_1-1$, we have:

11 $$\begin{aligned} \begin{aligned} \mathcal {P}_{(i,N)} = \xi _1^i \mathcal {P}_{(S_1,F)} \ and \ \mathcal {P}_{(i,F)} = \xi _2^i \mathcal {P}_{(S_1,F)}, \end{aligned} \end{aligned}$$

where

$$\begin{aligned} \begin{aligned} \xi _1^i = \frac{c_2 \xi _1^{i+1} + c_3 c_4 \xi _{2}^{i+1}}{1-c_1c_4}, \ \xi _2^i = c_3 \xi _2^{i+1} + \frac{c_1c_2 \xi _1^{i+1} + c_1 c_3c_4 \xi _{2}^{i+1}}{1-c_1c_4}, \ \forall \ i=S_2+1,\ldots ,S_1-1, \end{aligned} \end{aligned}$$

and $$\xi _1^{S_1} = 1/c_1$$, $$\xi _2^{S_1}=1$$.

*Step 2.* Using equation (7), for $i=s_1+2,\ldots ,s_2$, and equation (3), we have:

12 $$\begin{aligned} \mathcal {P}_{(i,N)} = \Psi _{i}\left(\mathcal {P}_{(s_1+1,N)},\mathcal {P}_{(0,F)}\right) = \left(\frac{1}{c_2}\right)^{i-s_1-1} \mathcal {P}_{(s_1+1,N)} - \left(\frac{\nu }{\lambda }\right)^{2} \sum _{j=s_1+1}^{i-1} \left(\frac{1}{c_2}\right)^{i-j-1} \left(\frac{1}{c_3}\right)^{j-1} \mathcal {P}_{(0,F)}. \end{aligned}$$

*Step 3.* Considering the sets of equations given in (4) and (7) together, for $i=s_1+1, s_1+2,\ldots ,S_2-1$, we have:

13 $$\begin{aligned} \mathcal {P}_{(i,N)} = \frac{c_2 \mathcal {P}_{(i+1,N)} +c_3c_4 \mathcal {P}_{(i+1,F)}}{1-c_1c_4}, \end{aligned}$$

and for $$i=S_2-1,S_2-2,\ldots ,s_2+1$$:

14 $$\begin{aligned} \mathcal {P}_{(i,F)} = c_3 \mathcal {P}_{(i+1,F)} + \frac{c_1c_2 \mathcal {P}_{(i+1,N)} +c_1c_3c_4 \mathcal {P}_{(i+1,F)}}{1-c_1c_4}, \end{aligned}$$

where $\mathcal {P}_{(S_2,N)}$ and $\mathcal {P}_{(S_2,F)}$ are obtained by equation (5) as follows:

15 $$\begin{aligned} \begin{aligned}&\mathcal {P}_{(S_2,N)} = \frac{1}{1-c_1} \Big (c_2 \xi _1^{S_2+1} + c_4 \xi _2^{S_2+1} \Big ) \mathcal {P}_{(S_1,F)} + c_1 \Big ( \mathcal {P}_{(s_1+1,N)} + \sum _{i=s_1+2}^{s_2} \Psi _{i}\big (\mathcal {P}_{(s_1+1,N)},\mathcal {P}_{(0,F)}\big ) \Big ). \\ {}&\mathcal {P}_{(S_2,F)} = \frac{1}{c_4} \Big (\mathcal {P}_{(S_2,N)} - c_2 \xi _1^{S_2+1} \mathcal {P}_{(S_1,F)} \Big ). \end{aligned} \end{aligned}$$

*Step 4.* Find $\mathcal {P}_{(s_1+1,N)}$ with respect to (w.r.t) $\mathcal {P}_{(S_1,F)}$ and $\mathcal {P}_{(0,F)}$ by solving the recursive equations in (13). Substituting the resulting expression in equation (8), we can find $\mathcal {P}_{(S_1,F)}$ w.r.t $\mathcal {P}_{(0,F)}$. Then, using equations (11)-(15) one can exploit $\mathcal {P}_{(i,F)}$ and $\mathcal {P}_{(i,N)}$ for all *i*’s w.r.t $\mathcal {P}_{(0,F)}$. Finally, equalizing the sum of all steady-state probabilities, which are expressed in terms of $\mathcal {P}_{(0,F)}$, to one (via equation (9)), we obtain $\mathcal {P}_{(0,F)}$ and consequently the other steady-state probability values.

Utilizing the algorithm described above, we obtain properties regarding the steady-state probabilities of inventory levels. The results are presented with the propositions below.

#### Proposition 1

In the $(s_1,S_1,s_2,S_2)$ policy, $\pi _{s_2+1}=\pi _{s_2+2}=\cdots =\pi _{S_2}$ at the steady state.

#### Proposition 2

In the $(s_1,S_1,s_2,S_2)$ policy, $\pi _{S_2+1}=\pi _{S_2+2}=\cdots =\pi _{S_1}$ at the steady state.

$\sum _{i=0}^{s_2} \pi _i = 1-\pi _{S_2}-\pi _{S_1}$ immediately follows from the results in Propositions 1 and 2. We use this relation together with the ones in the propositions to ease the calculations of some of the operating characteristics as discussed in the next subsection (e.g., see expression (17)).

### Operating characteristics

We use the stationary distribution of the inventory level derived in the previous section to formulate the operating characteristics of the system. The total expected cost consists of inventory holding, lost sales, and regular and emergency ordering costs. We denote the expected on-hand inventory and the expected number of lost sales by $\mathbb {E}[OH]$ and $\mathbb {E}[LS]$, respectively. $\mathbb {E}[RO]$ and $\mathbb {E}[EO]$ are used to express the expected regular and emergency ordering costs, respectively. Recall that $\pi _j$ is the fraction of time in the long run that the system holds *j* items on stock.

For the $S_2 \le S_1$ case, the system can hold at most $S_1$ items on hand. Hence, the expected on-hand inventory is given by

16 $$\begin{aligned} \mathbb {E}[OH] = \sum _{j=1}^{S_1} j \pi _j. \end{aligned}$$

Using the results in Propositions 1 and 2, $\mathbb {E}[OH]$ can be simplified as

17 $$\begin{aligned} \mathbb {E}[OH] = \sum _{j=1}^{s_2} j \pi _j + [(s_2+1)+(s_2+2)+\cdots +S_2] \pi _{S_2}+ [(S_2+1)+(S_2+2)+\cdots +S_1] \pi _{S_1}. \end{aligned}$$

$\pi _0$ is the fraction of the time during which the system is out of stock. Following, the expected number of lost sales is expressed as

18 $$\begin{aligned} \mathbb {E}[LS] = \lambda \pi _0. \end{aligned}$$

A regular order is placed in two cases, which can be summarized as follows. (1) If the inventory level is below $s_1$ (say $j \le s_1$) at the instant the supplier switches to *on*, a regular order of size $S_1-j$ is placed. Then, the inventory level transitions from state $j ~(\le s_1)$ to $S_1$ with rate $\nu$. (2) If a demand arrives during *on* period while the inventory level is $s_1+1$, a regular order of size $S_1-s_1$ is placed. Then, the inventory level switches from state $s_1$ to $S_1$ with rate $\lambda$. In both cases, a fixed cost $K_o$ and ordering cost of $c_o$ per unit are incurred. Accordingly, the expected regular ordering cost is expressed as

19 $$\begin{aligned} \mathbb {E}[RO] = \sum _{j=0}^{s_1} \nu [K_o + c_o(S_1-j)] \times \mathcal {P}_{(j,F)} + \lambda [K_o + c_o(S_1-s_1)] \times \mathcal {P}_{(s_1+1,N)}. \end{aligned}$$

An emergency order is placed to bring the inventory level up to level $S_2$ when the supplier switches to *off* mode and the inventory level is between $s_1$ and $s_2$ (say $s_1+1 \le j \le s_2$). This event occurs with rate $\mu$. The expected emergency ordering cost is given by

20 $$\begin{aligned} \mathbb {E}[EO] = \sum _{j=s_1+1}^{s_2} \mu [K_e + c_e(S_2-j)] \times \mathcal {P}_{(j,N)}. \end{aligned}$$

Note that for the case where $S_2 > S_1$, the derivation of explicit expressions for $\mathbb {E}[OH]$, $\mathbb {E}[LS]$, $\mathbb {E}[RO]$, and $\mathbb {E}[EO]$ can be treated similarly.

Using the operating characteristics, the expected total cost, $\mathbb {E}[C]$, can be written as:

21 $$\begin{aligned} \mathbb {E}[C] = \mathbb {E}[RO] + \mathbb {E}[EO] + h \mathbb {E}[OH] + b \mathbb {E}[LS]. \end{aligned}$$

As long as the limiting distribution of inventory level is known (for both $S_2 > S_1$ and $S_2 \le S_1$ cases), the optimal policy parameters $(s_1,S_1,s_2,S_2)$ can be found by solving the following mixed-integer nonlinear optimization problem:

$$\begin{aligned} \text {(P)}:&\min _{s_1,S_1,s_2,S_2} \mathbb {E}[C] \\ \text {subject to:}&\,s_1 \le s_2\\&s_2 \le S_2 \\&s_1 < S_1 \\&s_1,S_1,s_2,S_2 \in \mathbb {I}^+, \end{aligned}$$

where $\mathbb {I}^+$ is the set of nonnegative integer numbers. The objective of the problem (P) is to minimize the expected total cost subject to constraints showing the relations among the policy parameters. Note that we do not impose any relation between $S_1$ and $S_2$. It is important to highlight that one needs to optimize $S_2 \le S_1$ and $S_2>S_1$ cases separately. The one that returns the minimum expected cost should be considered as the optimal solution.

CTMC model of (*s*, *S*) policy and the derivation of the steady-state probabilities are provided in "Appendix 2." Similar to Propositions 1 and 2, one can easily show the following result for the (*s*, *S*) policy.

#### Proposition 3

In the (*s*, *S*) policy, at the steady state, $\pi _{s+1}=\pi _{s+2}=\cdots =\pi _{S}$.

### Solution approach

The optimization problem (P) is a mixed-integer nonlinear programming model, which makes it challenging to derive explicit expressions for the optimal policy parameters $(s_1^*, S_1^*, s_2^*, S_2^*)$. Therefore, we apply an enumeration search on all decision variables in a large search space to find their optimal values. $s_1$ and $s_2$ are bounded above by $S_1$ and $S_2$, respectively. We define an arbitrary upper bound, $\overline{S}$, for the regular order-up-to levels $S_1$ and $S_2$. When $S_1$ or $S_2$ hits the upper bound $\overline{S}$, we increase $\overline{S}$ to an arbitrary larger value. This step is repeated until no binding is observed.

After investigating the results of the enumeration search, we conjecture that $\mathbb {E}[C(s_1^*(S_1),S_1,s_2^*(S_1),S_2^*(S_1))]$, where $s_1^*(S_1)$, $s_2^*(S_1)$, and $S_2^*(S_1)$ are the optimal values for a given $S_1$, is convex with respect to $S_1$.

#### Conjecture 1

The smallest $S_1$ that satisfies the following inequality determines the optimal $S_1$ together with $s_1$, $s_2$, and $S_2$.

22 $$\begin{aligned} \mathbb {E}[C(s_1^*(S_1+1),S_1+1,s_2^*(S_1+1),S_2^*(S_1+1))] - \mathbb {E}[C(s_1^*(S_1),S_1,s_2^*(S_1),S_2^*(S_1))] \ge 0. \end{aligned}$$

**Fig. 2:**
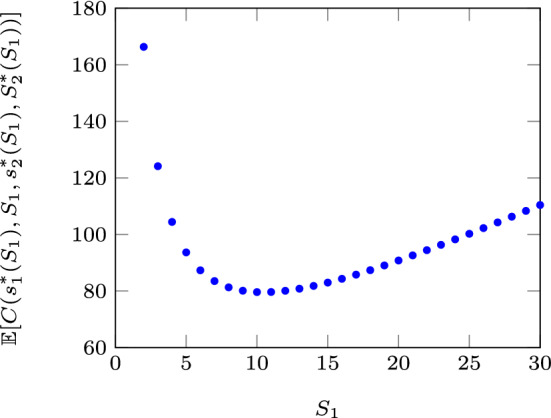
Convexity of the optimal expected cost rate w.r.t $S_1$ for $\lambda =5$, $h=5$, $b=100$, $c_o=5$, $c_e=8$, $K_o=K_e=50$, $\nu =5$, and $\mu =0.3$

An example showing the behavior of the optimal expected cost function with respect to $S_1$ is presented in Fig. 2. To plot this figure, the expected cost minimizing $s_1$, $S_2$, and $s_2$ are found for each $S_1$ value through a complete enumeration, and then the corresponding expected cost values are calculated.

In our numerical experiments, we utilize Conjecture 1 to reduce the search space of the enumeration algorithm. We set a large value for $\overline{S}$ and implement the enumeration algorithm to find the optimal solution.

## CTMC model of $(s_1,S_1,s_2,S_2)$ policy with exponentially distributed regular replenishment lead time

In this section, we extend our model in Sect. 3.2 by considering exponentially distributed regular and zero emergency lead times. As in Sect. 3, we first build the CTMC model of this new setting which yields the stationary distribution of the inventory level. Then, the resulting distribution is used to obtain the expected total operating cost of the system.

Before proceeding with the modeling, we describe how $(s_1,S_1,s_2,S_2)$ policy operates in this setting. If the inventory level hits $s_1$ when the supplier is *on*, a regular order of size $S_1-s_1$ is placed. The order is delivered after a lead time having an exponential distribution with rate $\ell$. When the regular lead time is nonzero, supplier’s availability does not only affect the acceptance of the orders but also the processing of the accepted (outstanding) order. Switching from *on* to *off* mode prevents the supplier from accepting orders. Also, the processing of any outstanding order is halted for the duration of the *off* period and it is resumed when the supplier turns back into *on* mode. This assumption is consistent with the idea of an unreliable supplier with random interruptions, as the supplier becomes fully unavailable during the *off* period. Moreover, we follow the same assumption as in the zero lead time case by allowing at most one order to be outstanding. This implies that the supplier cannot accept emergency orders during regular replenishment lead time. The retailer can place an emergency order at the beginning of the *off* period only if there is no outstanding order and the inventory level is below $s_2$. Besides, the relations among the policy parameters also hold here with the same reasoning as in the zero regular lead time case. Specifically, $s_1 \le s_2$, $s_1 < S_1$, and $s_2 \le S_2$.

For inventory models with lost sales, it is a typical approach to restrict outstanding orders by one to achieve analytical tractability (Gupta 1996; Parlar 1997; Mohebbi and Hao 2006, 2008). Following, we provide an example showing the complexity of analytical treatment of requesting an emergency order during the replenishment lead time (i.e., allowing for multiple outstanding orders). On the contrary, assume that multiple outstanding orders are allowed. In such a case, when the retailer switches from *on* to *off* mode, an emergency order is triggered in two cases: (1) when there is an outstanding regular order (since the inventory level $i\le s_1 \le s_2$), and (2) when there is no outstanding regular order and the inventory level is less than $s_2$. However, we do not know the probabilities of having cases (1) and (2). So, it makes the analysis intractable with the current state definition. If we include outstanding orders to the state definition, then we face dimensionality issues. We have a similar complexity in the analysis if we assume that the retailer can receive an order if the supplier is at *off* mode.

**Fig. 5:**
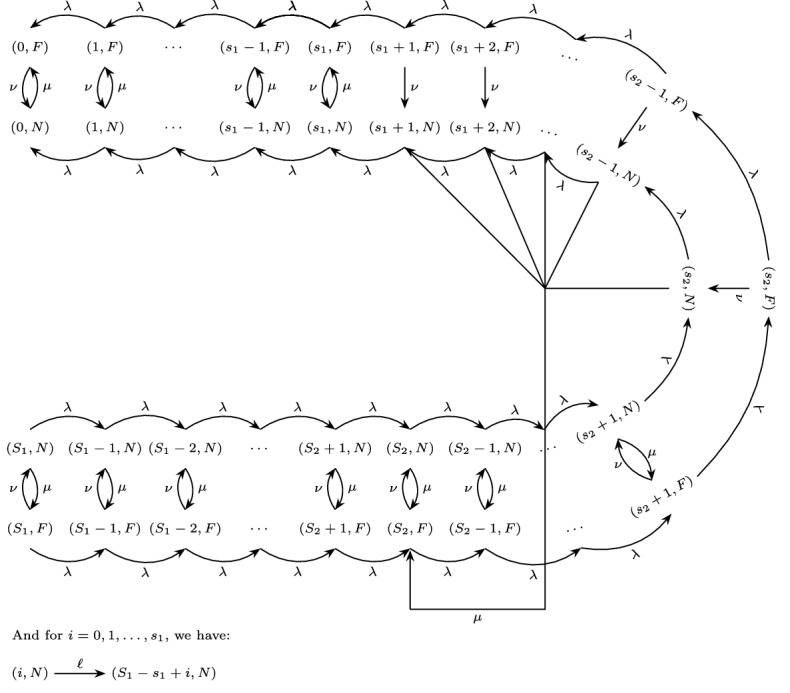
Markov chain transition diagram for the $(s_1,S_1,s_2,S_2)$ policy with exponential lead time

The transition rates and transition diagram of this new setting are presented in Table 3 and Fig. 5 in "Appendix 3," respectively. Note that the transition types 1 and 6 in Table 3 differ from the ones given for zero lead time case in Table 2.

**Table 3:** Transition rates of CTMC for exponential lead time

	From	To	Rate	Range
1	(*i*, *F*)	(*i*, *N*)	$\nu$	$i=0,1,\ldots ,S_1$
2	(*i*, *N*)	(*i*, *F*)	$\mu$	$i=0,1,\ldots ,s_1$ and $i=s_2+1,\ldots ,S_1$
3	(*i*, *N*)	$(S_2,F)$	$\mu$	$i=s_1+1,\ldots ,s_2$
4	(*i*, *F*)	$(i-1,F)$	$\lambda$	$i=1,2,\ldots ,S_1$
5	(*i*, *N*)	$(i-1,N)$	$\lambda$	$i=1,2,\ldots ,S_1$
6	(*i*, *N*)	$(S_1-s_1+i,N)$	$\ell$	$i=0,1,\ldots ,s_1$

We again report the analysis of $S_2 \le S_1$ case and the other case can be treated similarly. The balance equations of the new CTMC, for $S_2 \le S_1$, become:

23 $$\begin{aligned} \begin{aligned}&(1) \ \nu \mathcal {P}_{(0,F)} = \lambda \mathcal {P}_{(1,F)} + \mu \mathcal {P}_{(0,N)}. \\&(2) \ (\nu +\lambda ) \mathcal {P}_{(i,F)} = \lambda \mathcal {P}_{(i+1,F)} + \mu \mathcal {P}_{(i,N)}, \ \forall \ i=1,\ldots ,s_1 \ and \ i=s_2+1,\ldots ,S_2-1,S_2+1,\ldots ,S_1-1. \\&(3) \ (\nu +\lambda ) \mathcal {P}_{(i,F)} = \lambda \mathcal {P}_{(i+1,F)}, \ \forall \ i=s_1+1,\ldots ,s_2. \\ {}&(4) \ (\nu +\lambda ) \mathcal {P}_{(S_2,F)} = \lambda \mathcal {P}_{(S_2+1,F)} + \mu \Big (\sum _{i=s_1+1}^{s_2}\mathcal {P}_{(i,N)}+\mathcal {P}_{(S_2,N)}\Big ). \\ {}&(5) \ (\nu +\lambda ) \mathcal {P}_{(S_1,F)} = \mu \mathcal {P}_{(S_1,N)}. \\ {}&(6) \ (\mu +\ell ) \mathcal {P}_{(0,N)} = \nu \mathcal {P}_{(0,F)}+ \lambda \mathcal {P}_{(1,N)}. \\ {}&\text {If}\quad s_1<S_1-s_1: \\ {}&(7) \ (\mu +\lambda +\ell ) \mathcal {P}_{(i,N)} = \lambda \mathcal {P}_{(i+1,N)} + \nu \mathcal {P}_{(i,F)}, \ \forall \ i=1,2,\ldots ,s_1. \\ {}&(8) \ (\mu +\lambda ) \mathcal {P}_{(i,N)} = \lambda \mathcal {P}_{(i+1,N)} + \nu \mathcal {P}_{(i,F)}, \ \forall \ i=s_1+1,\ldots ,S_1-s_1-1. \\ {}&(9) \ (\mu +\lambda ) \mathcal {P}_{(i,N)} = \lambda \mathcal {P}_{(i+1,N)} + \nu \mathcal {P}_{(i,F)} + \ell \mathcal {P}_{(i-S_1+s_1,N)}, \ \forall \ i=S_1-s_1,\ldots ,S_1-1. \\ {}&\text {If}\quad s_1 \ge S_1-s_1: \\ {}&(7) \ (\mu +\lambda ) \mathcal {P}_{(i,N)} = \lambda \mathcal {P}_{(i+1,N)} + \nu \mathcal {P}_{(i,F)}, \ \forall \ i=1,2,\ldots ,S_1-s_1-1. \\ {}&(8) \ (\mu +\lambda + \ell ) \mathcal {P}_{(i,N)} = \lambda \mathcal {P}_{(i+1,N)} + \nu \mathcal {P}_{(i,F)}+ \ell \mathcal {P}_{(i-S_1+s_1,N)}, \ \forall \ i=S_1-s_1,\ldots ,s_1. \\ {}&(9) \ (\mu +\lambda ) \mathcal {P}_{(i,N)} = \lambda \mathcal {P}_{(i+1,N)} + \nu \mathcal {P}_{(i,F)}, \ \forall \ i=s_1+1,\ldots ,S_1-1. \\ {}&(10) \ (\mu +\lambda ) \mathcal {P}_{(S_1,N)} = \nu \mathcal {P}_{(S_1,F)} +\ell \mathcal {P}_{(s_1,N)}. \end{aligned} \end{aligned}$$

Next, we apply a similar methodology as in Sect. 3 to derive the expected total cost of operating $(s_1,S_1,s_2,S_2)$ policy. To that end, one can show that the new Markov chain presented in Fig. 5 is ergodic, which ensures that steady-state probabilities exist. A similar algorithm with the one given in Sect. 3.2 is utilized to find steady-state probabilities. The steady-state probabilities of inventory levels can be characterized as in Propositions 4 and 5. The proofs are not repeated due to space limitations.

### Proposition 4

At the steady state, $\pi _{s_2+1}=\pi _{s_2+2}=\cdots =\pi _{S_2}$.

### Proposition 5

At the steady state, $\pi _{S_2+1}=\pi _{S_2+2}=\cdots =\pi _{S_1-s_1}$.

One can see that the expected on hand inventory and the expected number of lost sales are given by Equations (17) and (18), respectively. Also, the expected emergency ordering cost is given by Equation (20). However, the expected regular ordering cost is reformulated as

24 $$\begin{aligned} \mathbb {E}[RO^{\prime }] = \sum _{j=0}^{s_1} \ell [K_o + c_o(S_1-s_1)] \times \mathcal {P}_{(j,N)}. \end{aligned}$$

Then, the expected total operating cost becomes

25 $$\begin{aligned} \mathbb {E}[C^{\prime }] = \mathbb {E}[RO^{\prime }] +\mathbb {E}[EO]+h\mathbb {E}[OH]+b\mathbb {E}[LS]. \end{aligned}$$

Here, we again conjecture that $\mathbb {E}[C^{\prime }]$ is convex w.r.t $S_1$ based on our numerical analysis. To find the optimal policy parameters, we implement the enumeration algorithm explained in Sect. 3.4 by considering the new expected cost function in (25).

It is important to point out that our analytical framework and solution approach can be easily applied to a setting with backordering. In that case, one needs to relax the nonnegativity constraint on $s_1$, while keeping the one for $s_2$ (i.e., $s_1 \in \mathbb {I}$ and $s_2,S_1,S_2 \in \mathbb {I}^+$). Nonnegative $s_2$ guarantees that the emergency order quantity can never be more than $S_2$. Thus, the zero emergency lead time assumption stays reasonable for this case as well.

## Numerical study

In this section, we develop a numerical study to illustrate the benefits of exercising $(s_1,S_1,s_2,S_2)$ policy instead of (*s*, *S*) policy when the supply is subject to random interruptions. The numerical results also provide insights into the value of disruption information as it gives the retailer an emergency ordering opportunity when the supply is disrupted. We also aim to identify the conditions under which $(s_1,S_1,s_2,S_2)$ policy brings considerable cost savings. Thus, we investigate the impact of different problem parameters on the optimal expected total costs, order-up-to levels, and savings achieved by $(s_1,S_1,s_2,S_2)$ policy.

We define a base data set by considering the parameter sets used in Berk and Arreola-Risa (1994) and Moinzadeh and Nahmias (1988). The numerical values for the base data set are displayed in Table 4. For emergency ordering related costs, we use the same setting with Moinzadeh and Nahmias (1988), where the ratio of $K_e/K_o$ is taken as 3, and $c_o$ and $c_e$ are both set to 5. $1/\nu$ and $\mu /\nu$ are selected at various levels in order to demonstrate the impact of disruption profile on performance of the $(s_1,S_1,s_2,S_2)$ policy. Note that $\mu /\nu$ determines the long run average time in which the supplier is available. The supplier’s availability can be found by the ratio $\frac{\mu }{\mu +\nu }$. Given mean length of the *off* period, the supplier’s availability corresponding to $\mu /\nu$ values of $\{ 1, 0.8, 0.5, 0.25, 0.1, 0.05, 0.01 \}$ are $\{ 50\%, 56\%,67\%, 80\%, 91\%, 95\%, 99\% \}$, respectively. For a specific $1/\nu$ value, supplier’s availability and expected length of the *on* period ($1/\mu$) increase as $\mu /\nu$ decreases.

**Table 4:** Base parameter set

Parameter	Notation	Value
Fixed cost of regular ordering	$K_o$	10
Fixed cost of emergency ordering	$K_e/K_o$	3
Regular ordering cost/unit	$c_o$	5
Emergency ordering cost/unit	$c_e$	5
Inventory holding cost	*h*	1
Lost sales cost	*b*	10
Demand rate	$\lambda$	5
Mean length of *off* period	$1/\nu$	10, 1, 0.5, 0.25, 0.1
Ratio of mean length of *off* period to and *on* period	$\mu /\nu$	1, 0.8, 0.5, 0.25, 0.1, 0.05, 0.01

As we are interested in benefits of exercising $(s_1,S_1,s_2,S_2)$ policy, we compare the system applying optimal $(s_1,S_1,s_2,S_2)$ policy with the one where optimal (*s*, *S*) policy is employed. Under the (*s*, *S*) policy, the stock is controlled as follows: If the inventory level is at or below *s* and the supply is available, place an order to bring the inventory level up to *S* and do not place an order otherwise. Appendices A2 and B2 present the models to calculate the optimal *s* and *S*, and the optimal expected total cost for zero and exponential regular lead times, respectively. The numerical results for both of the inventory policies are validated through simulation (Please see "Appendix 6" for simulation details). We use percentage improvement in the expected cost due to implementing the $(s_1,S_1,s_2,S_2)$ policy against the (*s*, *S*) policy as our performance measure, which is given by

26 $$\begin{aligned} \% \text{ Gap}_{12} = \frac{E[C_2]-E[C_1]}{E[C_2]} \times 100 , \end{aligned}$$

where $E[C_1]$ is the expected cost of $(s_1,S_1,s_2,S_2)$ policy and $E[C_2]$ is the expected cost of (*s*, *S*) policy.

As a result of the numerical experiments, we report the optimal policy parameters and the expected total cost for $(s_1,S_1,s_2,S_2)$ and (*s*, *S*) policies, and the percentage savings achieved via exercising $(s_1,S_1,s_2,S_2)$ policy instead of the (*s*, *S*) policy.

We conduct three sets of analyses with different regular replenishment lead time assumptions: (1) zero, (2) positive constant, and (3) exponentially distributed. We start our discussion with zero regular replenishment lead time case. We first analyze results of the base parameter set and then conduct sensitivity analysis with respect to different parameters. Sensitivity analysis done with positive constant and exponentially distributed regular lead time assumptions has yielded similar qualitative results; thus, they will not be displayed.

### Numerical results of zero regular lead time case

In this subsection, we consider the setting with zero regular replenishment lead time. Computations are made based on the models and solution approaches given in Sect. 3 and "Appendix 3" for $(s_1,S_1,s_2,S_2)$ and (*s*, *S*) policies, respectively.

Table 5 demonstrates the results for the base parameter set. To capture the effect of different parameters on performance of polices, we use the base parameter set and conduct sensitivity analysis on *b* and $K_o$ values. Particularly, in Tables 5 through 8 we consider the values $(b,K_o) = (10 ,10)$, $(b,K_o) = (10 ,100)$, $(b,K_o) = (100, 10)$, $(b,K_o) = (100, 100)$, respectively. Also, we run the computations by setting $K_e/K_o$ to 5 for each data set in addition to the value in the base parameter set to incorporate the effect of fixed emergency ordering cost on the results. Recall that $(s_1,S_1,s_2,S_2)$ policy reduces to (*s*, *S*) policy either when $s_2=0$ or $s_2=S_2$.

After having a close look at the results, we first elaborate on the behavior of the optimal policy parameters and the resulting expected costs. Our observations are as follows.

- Unit lost sales cost plays a key role in determining the policy parameters. The optimal policy parameters, $s_1^*,S_1^*,s_2^*,S_2^*,s^*,S^*$, are non-decreasing and the associated expected costs of both policies show increasing behavior as the unit lost sales cost increases.
- $K_o$ is another important factor affecting policy parameters. Higher fixed cost enforces the retailer to order less frequently. Thus, order-up-to levels, $S_1^{*}$ and $S^{*}$, increase as $K_o$ increases from 10 to 100. A high value of $K_o$ corresponds to a high $K_e$ value. As $K_e$ increases, $S_2^*$ increases as well if emergency ordering is being utilized. Expected costs of the policies also increase with $K_o$ as expected.
- $1/\nu$ and $\mu /\nu$ characterize the distributions of *on* and *off* periods’ lengths. Changing these parameters has mixed impacts on the policy parameters. However, the expected cost of exercising both policies increases consistently as the supplier’s availability decreases (i.e., $\mu /\nu$ increases) for a given expected length of the *off* period.
- For the instances that use emergency orders, $s_1^*$ and $S_1^*$ are below $s^*$ and $S^*$, respectively. When there is no emergency ordering opportunity, the only way to respond to the disruption risk is to keep more inventory and it leads to higher reorder and order-up-to levels.

The emergency ordering opportunity at the disruption point enables us to hedge against shortages during the *off* period. This fact encourages the retailer to place an emergency order when the unit lost sales cost is high. Therefore, it is expected $(s_1,S_1,s_2,S_2)$ policy to perform better than (*s*, *S*) policy at a higher unit lost sales cost. The numerical results in Tables 5 through 8 confirm this expectation. When $b=10$, emergency ordering option is not used for the majority of the instances (see Tables 5 and 6).

Tables 7 and 8 indicate that mean length of *off* period ($1/\nu$) and supplier’s availability ($\frac{\nu }{\mu +\nu }$) have significant impacts on the policy parameters as well as on performance of the $(s_1,S_1,s_2,S_2)$ policy. Note that the supplier’s availability is increasing in $\mu /\nu$ for a given expected duration of the *off* period. The percentage improvement in the expected cost achieved via $(s_1,S_1,s_2,S_2)$ policy is more significant for high values of $1/\nu$ and $\mu /\nu$. This observation implies that emergency ordering opportunity is especially efficient when there is a potential for a long disruption period and the percentage of supplier’s availability is low. Moreover, the results show that the percentage improvement in the expected cost decreases as $K_e/K_o$ increases from 3 to 5. The benefit of emergency orders is the ability to account for the trade-off between lost sales cost reduction and emergency ordering cost. Therefore, the cost savings due to the emergency orders are larger as long as the fixed cost of emergency ordering is not getting too large with respect to the decrease in the unit lost sales cost. Even for high values of $K_e$, it might still yield benefits to exercise the $(s_1,S_1,s_2,S_2)$ policy if the expected duration of *off* period is long.

It is worth pointing out that our results are in line with an earlier related study. Bakal et al. (2017) investigated the value of additional disruption orders, while imposing stationary deterministic demand assumption and allowing for backorders. Their numerical study also indicated that the retailer may not choose the disruption order opportunity when the backordering cost is low and/or the expected length of the *off* period is short. Nevertheless, it should be recognized that the two studies apply to different settings. We focus on models that reflect a more realistic environment in terms of considering demand uncertainty and different lead time scenarios. In addition, it is noteworthy that the $(s_1,S_1,s_2,S_2)$ policy would yield even higher cost savings if parameters of the (*s*, *S*) policy are estimated without considering the disruption risk.

### Numerical results for positive constant regular replenishment lead time case

In this section, we analyze $(s_1,S_1,s_2,S_2)$ policy at a setting with positive lead time for regular ordering ($L_o$), while still taking the emergency shipment lead time as zero. As the analytical treatment of the model is quite complex under this setting, we only conduct a simulation study to assess the benefits of the emergency ordering option. In particular, we use simulation optimization, which is replicated ten times each with a run length of one million days and a warm-up period of 1000 days.

**Table 5:** Comparison of the (*s*, *S*) policy and $(s_1,S_1,s_2,S_2)$ policy when $K_o=10$ and $b=10$ ($\lambda =5$, $h=1$, $c_o=c_e=5$)

$K_e/K_o$	$1/\nu$	$\mu /\nu$	$(s_1^*,S_1^*,s_2^*,S_2^*)$	$\mathbb {E}[C_1]$	$(s^*,S^*)$	$\mathbb {E}[C_2]$	%Gap$_{12}$
3	10	1	(0, 11, 4, 20)	41.84	(0, 11)	41.95	0.26
		0.8	(0, 11, 4, 20)	41.15	(0, 11)	41.25	0.24
		0.5	(0, 11, 4, 20)	39.75	(0, 11)	39.82	0.19
		0.25	(0, 10, 4, 20)	38.05	(0, 10)	38.10	0.13
		0.1	(0, 10, 4, 20)	36.66	(0, 10)	36.68	0.06
		0.05	(0, 10, 3, 20)	36.11	(0, 10)	36.12	0.03
		0.01	(0, 10, 3, 20)	35.63	(0, 10)	35.63	0.01
	1	1	(0, 12, 0, 28)	38.10	(0, 12)	38.10	0.00
		0.8	(0, 12, 0, 22)	37.86	(0, 12)	37.86	0.00
		0.5	(0, 12, 0, 22)	37.35	(0, 12)	37.35	0.00
		0.25	(0, 11, 0, 33)	36.65	(0, 11)	36.65	0.00
		0.1	(0, 10, 0, 34)	36.05	(0, 10)	36.05	0.00
		0.05	(0, 10, 0, 40)	35.79	(0, 10)	35.79	0.00
		0.01	(0, 10, 0, 31)	35.56	(0, 10)	35.56	0.00
	0.5	1	(0, 12, 0, 28)	37.02	(0, 12)	37.02	0.00
		0.8	(0, 11, 0, 34)	36.87	(0, 11)	36.87	0.00
		0.5	(0, 11, 0, 40)	36.56	(0, 11)	36.56	0.00
		0.25	(0, 11, 0, 36)	36.17	(0, 11)	36.17	0.00
		0.1	(0, 10, 0, 41)	35.81	(0, 10)	35.81	0.00
		0.05	(0, 10, 0, 46)	35.67	(0, 10)	35.67	0.00
		0.01	(0, 10, 0, 34)	35.53	(0, 10)	35.53	0.00
	0.25	1	(0, 11, 0, 19)	36.32	(0, 11)	36.32	0.00
		0.8	(0, 11, 0, 15)	36.24	(0, 11)	36.24	0.00
		0.5	(0, 11, 0, 15)	36.07	(0, 11)	36.07	0.00
		0.25	(0, 10, 0, 20)	35.85	(0, 10)	35.85	0.00
		0.1	(0, 10, 0, 20)	35.66	(0, 10)	35.66	0.00
		0.05	(0, 10, 0, 17)	35.59	(0, 10)	35.59	0.00
		0.01	(0, 10, 0, 11)	35.52	(0, 10)	35.52	0.00
	0.1	1	(0, 10, 0, 22)	35.85	(0, 10)	35.85	0.00
		0.8	(0, 10, 0, 42)	35.82	(0, 10)	35.82	0.00
		0.5	(0, 10, 0, 27)	35.74	(0, 10)	35.74	0.00
		0.25	(0, 10, 0, 46)	35.64	(0, 10)	35.64	0.00
		0.1	(0, 10, 0, 39)	35.57	(0, 10)	35.57	0.00
		0.05	(0, 10, 0, 28)	35.53	(0, 10)	35.53	0.00
		0.01	(0, 10, 0, 20)	35.51	(0, 10)	35.51	0.00
5	10	1	(0, 11, 0, 0)	41.95	(0, 11)	41.95	0.00
		0.8	(0, 11, 0, 0)	41.25	(0, 11)	41.25	0.00
		0.5	(0, 11, 0, 0)	39.82	(0, 11)	39.82	0.00
		0.25	(0, 10, 0, 0)	38.10	(0, 10)	38.10	0.00
		0.1	(0, 10, 0, 0)	36.68	(0, 10)	36.68	0.00
		0.05	(0, 10, 0, 0)	36.12	(0, 10)	36.12	0.00
		0.01	(0, 10, 0, 0)	35.63	(0, 10)	35.63	0.00
	1	1	(0, 12, 0, 0)	38.10	(0, 12)	38.10	0.00
		0.8	(0, 12, 0, 0)	37.86	(0, 12)	37.86	0.00
		0.5	(0, 12, 0, 0)	37.35	(0, 12)	37.35	0.00
		0.25	(0, 11, 0, 0)	36.65	(0, 11)	36.65	0.00
		0.1	(0, 10, 0, 0)	36.05	(0, 10)	36.05	0.00
		0.05	(0, 10, 0, 0)	35.79	(0, 10)	35.79	0.00
		0.01	(0, 10, 0, 0)	35.56	(0, 10)	35.56	0.00
	0.5	1	(0, 12, 0, 0)	37.02	(0, 12)	37.02	0.00
		0.8	(0, 11, 0, 0)	36.87	(0, 11)	36.87	0.00
		0.5	(0, 11, 0, 0)	36.56	(0, 11)	36.56	0.00
		0.25	(0, 11, 0, 0)	36.17	(0, 11)	36.17	0.00
		0.1	(0, 10, 0, 0)	35.81	(0, 10)	35.81	0.00
		0.05	(0, 10, 0, 0)	35.67	(0, 10)	35.67	0.00
		0.01	(0, 10, 0, 0)	35.53	(0, 10)	35.53	0.00
	0.25	1	(0, 11, 0, 0)	36.32	(0, 11)	36.32	0.00
		0.8	(0, 11, 0, 0)	36.24	(0, 11)	36.24	0.00
		0.5	(0, 11, 0, 0)	36.07	(0, 11)	36.07	0.00
		0.25	(0, 10, 0, 0)	35.85	(0, 10)	35.85	0.00
		0.1	(0, 10, 0, 0)	35.66	(0, 10)	35.66	0.00
		0.05	(0, 10, 0, 0)	35.59	(0, 10)	35.59	0.00
		0.01	(0, 10, 0, 0)	35.52	(0, 10)	35.52	0.00
	0.1	1	(0, 10, 0, 0)	35.85	(0, 10)	35.85	0.00
		0.8	(0, 10, 0, 0)	35.82	(0, 10)	35.82	0.00
		0.5	(0, 10, 0, 0)	35.74	(0, 10)	35.74	0.00
		0.25	(0, 10, 0, 0)	35.64	(0, 10)	35.64	0.00
		0.1	(0, 10, 0, 0)	35.57	(0, 10)	35.57	0.00
		0.05	(0, 10, 0, 0)	35.53	(0, 10)	35.53	0.00
		0.01	(0, 10, 0, 0)	35.51	(0, 10)	35.51	0.00

**Table 6:** Comparison of the (*s*, *S*) policy and $(s_1,S_1,s_2,S_2)$ policy when $K_o=100$ and $b=10$ ($\lambda =5$, $h=1$, $c_o=c_e=5$)

$K_e/K_o$	$1/\nu$	$\mu /\nu$	$(s_1^*,S_1^*,s_2^*,S_2^*)$	$\mathbb {E}[C_1]$	$(s^*,S^*)$	$\mathbb {E}[C_2]$	%Gap$_{12}$
3	10	1	(0, 30, 0, 0)	54.55	(0, 30)	54.55	0
		0.8	(0, 31, 0, 0)	54.83	(0, 31)	54.83	0
		0.5	(0, 31, 0, 0)	55.39	(0, 31)	55.39	0
		0.25	(0, 31, 0, 0)	56.08	(0, 31)	56.08	0
		0.1	(0, 31, 0, 0)	56.65	(0, 31)	56.65	0
		0.05	(0, 32, 0, 0)	56.88	(0, 32)	56.88	0
		0.01	(0, 32, 0, 0)	57.07	(0, 32)	57.07	0
	1	1	(0, 31, 0, 0)	56.60	(0, 31)	56.60	0
		0.8	(0, 31, 0, 0)	56.65	(0, 31)	56.65	0
		0.5	(0, 31, 0, 0)	56.77	(0, 31)	56.77	0
		0.25	(0, 31, 0, 0)	56.91	(0, 31)	56.91	0
		0.1	(0, 32, 0, 0)	57.03	(0, 32)	57.03	0
		0.05	(0, 32, 0, 0)	57.07	(0, 32)	57.07	0
		0.01	(0, 32, 0, 0)	57.11	(0, 32)	57.11	0
	0.5	1	(0, 31, 0, 0)	56.85	(0, 31)	56.85	0
		0.8	(0, 31, 0, 0)	56.88	(0, 31)	56.88	0
		0.5	(0, 31, 0, 0)	56.94	(0, 31)	56.94	0
		0.25	(0, 32, 0, 0)	57.02	(0, 32)	57.02	0
		0.1	(0, 32, 0, 0)	57.07	(0, 32)	57.07	0
		0.05	(0, 32, 0, 0)	57.10	(0, 32)	57.10	0
		0.01	(0, 32, 0, 0)	57.12	(0, 32)	57.12	0
	0.25	1	(0, 31, 0, 0)	56.99	(0, 31)	56.99	0
		0.8	(0, 32, 0, 0)	57.00	(0, 32)	57.00	0
		0.5	(0, 32, 0, 0)	57.03	(0, 32)	57.03	0
		0.25	(0, 32, 0, 0)	57.07	(0, 32)	57.07	0
		0.1	(0, 32, 0, 0)	57.10	(0, 32)	57.10	0
		0.05	(0, 32, 0, 0)	57.11	(0, 32)	57.11	0
		0.01	(0, 32, 0, 0)	57.12	(0, 32)	57.12	0
	0.1	1	(0, 32, 0, 0)	57.07	(0, 32)	57.07	0
		0.8	(0, 32, 0, 0)	57.08	(0, 32)	57.08	0
		0.5	(0, 32, 0, 0)	57.09	(0, 32)	57.09	0
		0.25	(0, 32, 0, 0)	57.10	(0, 32)	57.10	0
		0.1	(0, 32, 0, 0)	57.11	(0, 32)	57.11	0
		0.05	(0, 32, 0, 0)	57.12	(0, 32)	57.12	0
		0.01	(0, 32, 0, 0)	57.12	(0, 32)	57.12	0
5	10	1	(0, 30, 0, 0)	54.55	(0, 30)	54.55	0
		0.8	(0, 31, 0, 0)	54.83	(0, 31)	54.83	0
		0.5	(0, 31, 0, 0)	55.39	(0, 31)	55.39	0
		0.25	(0, 31, 0, 0)	56.08	(0, 31)	56.08	0
		0.1	(0, 31, 0, 0)	56.65	(0, 31)	56.65	0
		0.05	(0, 32, 0, 0)	56.88	(0, 32)	56.88	0
		0.01	(0, 32, 0, 0)	57.07	(0, 32)	57.07	0
	1	1	(0, 31, 0, 0)	56.60	(0, 31)	56.60	0
		0.8	(0, 31, 0, 0)	56.65	(0, 31)	56.65	0
		0.5	(0, 31, 0, 0)	56.77	(0, 31)	56.77	0
		0.25	(0, 31, 0, 0)	56.91	(0, 31)	56.91	0
		0.1	(0, 32, 0, 0)	57.03	(0, 32)	57.03	0
		0.05	(0, 32, 0, 0)	57.07	(0, 32)	57.07	0
		0.01	(0, 32, 0, 0)	57.11	(0, 32)	57.11	0
	0.5	1	(0, 31, 0, 0)	56.85	(0, 31)	56.85	0
		0.8	(0, 31, 0, 0)	56.88	(0, 31)	56.88	0
		0.5	(0, 31, 0, 0)	56.94	(0, 31)	56.94	0
		0.25	(0, 32, 0, 0)	57.02	(0, 32)	57.02	0
		0.1	(0, 32, 0, 0)	57.07	(0, 32)	57.07	0
		0.05	(0, 32, 0, 0)	57.10	(0, 32)	57.10	0
		0.01	(0, 32, 0, 0)	57.12	(0, 32)	57.12	0
	0.25	1	(0, 31, 0, 0)	56.99	(0, 31)	56.99	0
		0.8	(0, 32, 0, 0)	57.00	(0, 32)	57.00	0
		0.5	(0, 32, 0, 0)	57.03	(0, 32)	57.03	0
		0.25	(0, 32, 0, 0)	57.07	(0, 32)	57.07	0
		0.1	(0, 32, 0, 0)	57.10	(0, 32)	57.10	0
		0.05	(0, 32, 0, 0)	57.11	(0, 32)	57.11	0
		0.01	(0, 32, 0, 0)	57.12	(0, 32)	57.12	0
	0.1	1	(0, 32, 0, 0)	57.07	(0, 32)	57.07	0
		0.8	(0, 32, 0, 0)	57.08	(0, 32)	57.08	0
		0.5	(0, 32, 0, 0)	57.09	(0, 32)	57.09	0
		0.25	(0, 32, 0, 0)	57.10	(0, 32)	57.10	0
		0.1	(0, 32, 0, 0)	57.11	(0, 32)	57.11	0
		0.05	(0, 32, 0, 0)	57.12	(0, 32)	57.12	0
		0.01	(0, 32, 0, 0)	57.12	(0, 32)	57.12	0

**Table 7:** Comparison of the (*s*, *S*) policy and $(s_1,S_1,s_2,S_2)$ policy when $K_o=10$ and $b=100$ ($\lambda =5$, $h=1$, $c_o=c_e=5$)

$K_e/K_o$	$1/\nu$	$\mu /\nu$	$(s_1^*,S_1^*,s_2^*,S_2^*)$	$\mathbb {E}[C_1]$	$(s^*,S^*)$	$\mathbb {E}[C_2]$	%Gap$_{12}$
3	10	1	(0, 10, 84, 97)	103.60	(70, 95)	135.48	23.53
		0.8	(0, 10, 83, 96)	96.71	(64, 90)	132.40	26.96
		0.5	(0, 10, 81, 93)	82.23	(49, 76)	123.58	33.46
		0.25	(0, 10, 80, 91)	63.99	(22, 51)	104.63	38.84
		0.1	(0, 10, 79, 90)	48.58	(0, 18)	72.28	32.79
		0.05	(0, 10, 78, 89)	42.37	(0, 13)	55.14	23.15
		0.01	(0, 10, 78, 89)	36.93	(0, 10)	39.63	6.81
	1	1	(14, 28, 0, 0)	53.28	(14, 28)	53.28	0.00
		0.8	(0, 26, 13, 27)	52.16	(14, 27)	52.94	1.47
		0.5	(0, 23, 12, 25)	49.27	(12, 26)	52.00	5.25
		0.25	(0, 9, 12, 23)	44.54	(9, 24)	49.97	10.86
		0.1	(0, 10, 12, 21)	39.83	(5, 20)	46.27	13.93
		0.05	(0, 10, 12, 21)	37.81	(1, 17)	42.98	12.05
		0.01	(0, 10, 12, 20)	35.99	(0, 11)	37.35	3.66
	0.5	1	(8, 20, 0, 0)	45.48	(8, 20)	45.48	0.00
		0.8	(8, 20, 0, 0)	45.29	(8, 20)	45.29	0.00
		0.5	(7, 19, 0, 0)	44.75	(7, 19)	44.75	0.00
		0.25	(0, 17, 6, 17)	42.44	(5, 18)	43.63	2.73
		0.1	(0, 13, 6, 15)	39.14	(3, 16)	41.58	5.87
		0.05	(0, 12, 5, 15)	37.54	(1, 14)	39.79	5.64
		0.01	(0, 10, 5, 14)	35.96	(0, 11)	36.56	1.66
	0.25	1	(5, 16, 0, 0)	41.27	(5, 16)	41.27	0.00
		0.8	(4, 16, 0, 0)	41.15	(4, 16)	41.15	0.00
		0.5	(4, 15, 0, 0)	40.77	(4, 15)	40.77	0.00
		0.25	(3, 15, 0, 0)	40.09	(3, 15)	40.09	0.00
		0.1	(0, 13, 2, 13)	38.34	(2, 13)	38.93	1.51
		0.05	(0, 12, 2, 12)	37.09	(1, 12)	37.90	2.14
		0.01	(0, 10, 2, 11)	35.86	(0, 11)	36.07	0.58
	0.1	1	(2, 13, 0, 0)	38.47	(2, 13)	38.47	0.00
		0.8	(2, 13, 0, 0)	38.37	(2, 13)	38.37	0.00
		0.5	(2, 13, 0, 0)	38.17	(2, 13)	38.17	0.00
		0.25	(1, 12, 0, 0)	37.87	(1, 12)	37.87	0.00
		0.1	(1, 12, 0, 0)	37.15	(1, 12)	37.15	0.00
		0.05	(0, 11, 0, 0)	36.55	(0, 11)	36.55	0.00
		0.01	(0, 10, 0, 0)	35.73	(0, 10)	35.73	0.00
5	10	1	(0, 10, 81, 98)	104.57	(70, 95)	135.48	22.82
		0.8	(0, 10, 80, 96)	97.57	(64, 90)	132.40	26.30
		0.5	(0, 10, 78, 94)	82.89	(49, 76)	123.58	32.93
		0.25	(0, 10, 77, 91)	64.38	(22, 51)	104.63	38.46
		0.1	(0, 10, 76, 90)	48.76	(0, 18)	72.28	32.54
		0.05	(0, 10, 75, 89)	42.47	(0, 13)	55.14	22.97
		0.01	(0, 10, 75, 89)	36.95	(0, 10)	39.63	6.76
	1	1	(14, 28, 0, 0)	53.28	(14, 28)	53.28	0.00
		0.8	(14, 27, 0, 0)	52.94	(14, 27)	52.94	0.00
		0.5	(12, 26, 0, 0)	52.00	(12, 26)	52.00	0.00
		0.25	(0, 20, 10, 24)	47.06	(9, 24)	49.97	5.81
		0.1	(0, 10, 10, 22)	41.45	(5, 20)	46.27	10.43
		0.05	(0, 10, 10, 21)	38.70	(1, 17)	42.98	9.96
		0.01	(0, 10, 10, 21)	36.18	(0, 11)	37.35	3.13
	0.5	1	(8, 20, 0, 0)	45.48	(8, 20)	45.48	0.00
		0.8	(8, 20, 0, 0)	45.29	(8, 20)	45.29	0.00
		0.5	(7, 19, 0, 0)	44.75	(7, 19)	44.75	0.00
		0.25	(5, 18, 0, 0)	43.63	(5, 18)	43.63	0.00
		0.1	(0, 15, 4, 16)	40.32	(3, 16)	41.58	3.03
		0.05	(0, 13, 4, 15)	38.26	(1, 14)	39.79	3.84
		0.01	(0, 11, 4, 14)	36.13	(0, 11)	36.56	1.17
	0.25	1	(5, 16, 0, 0)	41.27	(5, 16)	41.27	0.00
		0.8	(4, 16, 0, 0)	41.15	(4, 16)	41.15	0.00
		0.5	(4, 15, 0, 0)	40.77	(4, 15)	40.77	0.00
		0.25	(3, 15, 0, 0)	40.09	(3, 15)	40.09	0.00
		0.1	(2, 13, 0, 0)	38.93	(2, 13)	38.93	0.00
		0.05	(0, 12, 1, 12)	37.60	(1, 12)	37.90	0.80
		0.01	(0, 10, 1, 10)	35.98	(0, 11)	36.07	0.23
	0.1	1	(2, 13, 0, 0)	38.47	(2, 13)	38.47	0.00
		0.8	(2, 13, 0, 0)	38.37	(2, 13)	38.37	0.00
		0.5	(2, 13, 0, 0)	38.17	(2, 13)	38.17	0.00
		0.25	(1, 12, 0, 0)	37.87	(1, 12)	37.87	0.00
		0.1	(1, 12, 0, 0)	37.15	(1, 12)	37.15	0.00
		0.05	(0, 11, 0, 0)	36.55	(0, 11)	36.55	0.00
		0.01	(0, 10, 0, 0)	35.73	(0, 10)	35.73	0.00

**Table 8:** Comparison of the (*s*, *S*) policy and $(s_1,S_1,s_2,S_2)$ policy when $K_o=100$ and $b=100$ ($\lambda =5$, $h=1$, $c_o=c_e=5$)

$K_e/K_o$	$1/\nu$	$\mu /\nu$	$(s_1^*,S_1^*,s_2^*,S_2^*)$	$\mathbb {E}[C_1]$	$(s^*,S^*)$	$\mathbb {E}[C_2]$	%Gap$_{12}$
3	10	1	(0, 29, 66, 107)	121.38	(55, 110)	142.77	14.99
		0.8	(0, 29, 65, 105)	114.75	(49, 105)	139.85	17.95
		0.5	(0, 30, 64, 102)	100.94	(34, 94)	131.37	23.17
		0.25	(0, 31, 63, 99)	83.71	(7, 70)	112.79	25.78
		0.1	(0, 31, 63, 98)	69.29	(0, 44)	85.26	18.73
		0.05	(0, 31, 62, 97)	63.51	(0, 37)	72.28	12.13
		0.01	(0, 32, 62, 97)	58.46	(0, 33)	60.34	3.12
	1	1	(10, 44, 0, 0)	69.97	(10, 44)	69.97	0.00
		0.8	(9, 44, 0, 0)	69.61	(9, 44)	69.61	0.00
		0.5	(7, 43, 0, 0)	68.66	(7, 43)	68.66	0.00
		0.25	(5, 41, 0, 0)	66.59	(5, 41)	66.59	0.00
		0.1	(0, 37, 3, 37)	62.19	(0, 37)	62.88	1.10
		0.05	(0, 34, 3, 35)	59.88	(0, 35)	60.27	0.65
		0.01	(0, 32, 3, 33)	57.72	(0, 32)	57.81	0.16
	0.5	1	(5, 39, 0, 0)	64.12	(5, 39)	64.12	0.00
		0.8	(5, 38, 0, 0)	63.91	(5, 38)	63.91	0.00
		0.5	(4, 38, 0, 0)	63.35	(4, 38)	63.35	0.00
		0.25	(3, 37, 0, 0)	62.22	(3, 37)	62.22	0.00
		0.1	(0, 35, 0, 0)	60.14	(0, 35)	60.14	0.00
		0.05	(0, 33, 0, 0)	58.74	(0, 33)	58.74	0.00
		0.01	(0, 32, 0, 0)	57.47	(0, 32)	57.47	0.00
	0.25	1	(3, 36, 0, 0)	61.05	(3, 36)	61.05	0.00
		0.8	(3, 35, 0, 0)	60.95	(3, 35)	60.95	0.00
		0.5	(2, 35, 0, 0)	60.57	(2, 35)	60.57	0.00
		0.25	(1, 34, 0, 0)	59.89	(1, 34)	59.89	0.00
		0.1	(0, 33, 0, 0)	58.67	(0, 33)	58.67	0.00
		0.05	(0, 32, 0, 0)	57.95	(0, 32)	57.95	0.00
		0.01	(0, 32, 0, 0)	57.30	(0, 32)	57.30	0.00
	0.1	1	(1, 34, 0, 0)	59.10	(1, 34)	59.10	0.00
		0.8	(1, 33, 0, 0)	58.99	(1, 33)	58.99	0.00
		0.5	(1, 33, 0, 0)	58.78	(1, 33)	58.78	0.00
		0.25	(1, 33, 0, 0)	58.49	(0, 33)	58.49	0.00
		0.1	(1, 32, 0, 0)	57.75	(0, 32)	57.75	0.00
		0.05	(1, 32, 0, 0)	57.45	(0, 32)	57.45	0.00
		0.01	(1, 32, 0, 0)	57.19	(0, 32)	57.19	0.00
5	10	1	(0, 29, 58, 112)	129.27	(55, 110)	142.77	9.46
		0.8	(0, 29, 57, 109)	122.09	(49, 105)	139.85	12.71
		0.5	(0, 30, 55, 104)	106.84	(34, 94)	131.37	18.67
		0.25	(0, 31, 54, 100)	87.47	(7, 70)	112.79	22.45
		0.1	(0, 31, 54, 98)	71.07	(0, 44)	85.26	16.65
		0.05	(0, 31, 53, 97)	64.45	(0, 37)	72.28	10.83
		0.01	(0, 32, 53, 97)	58.65	(0, 33)	60.34	2.79
	1	1	(10, 44, 0, 0)	69.97	(10, 44)	69.97	0.00
		0.8	(9, 44, 0, 0)	69.61	(9, 44)	69.61	0.00
		0.5	(7, 43, 0, 0)	68.66	(7, 43)	68.66	0.00
		0.25	(5, 41, 0, 0)	66.59	(5, 41)	66.59	0.00
		0.1	(0, 37, 0, 0)	62.88	(0, 37)	62.88	0.00
		0.05	(0, 35, 0, 0)	60.27	(0, 35)	60.27	0.00
		0.01	(0, 32, 0, 0)	57.81	(0, 32)	57.81	0.00
	0.5	1	(5, 39, 0, 0)	64.12	(5, 39)	64.12	0.00
		0.8	(5, 38, 0, 0)	63.91	(5, 38)	63.91	0.00
		0.5	(4, 38, 0, 0)	63.35	(4, 38)	63.35	0.00
		0.25	(3, 37, 0, 0)	62.22	(3, 37)	62.22	0.00
		0.1	(0, 35, 0, 0)	60.14	(0, 35)	60.14	0.00
		0.05	(0, 33, 0, 0)	58.74	(0, 33)	58.74	0.00
		0.01	(0, 32, 0, 0)	57.47	(0, 32)	57.47	0.00
	0.25	1	(3, 36, 0, 0)	61.05	(3, 36)	61.05	0.00
		0.8	(3, 35, 0, 0)	60.95	(3, 35)	60.95	0.00
		0.5	(2, 35, 0, 0)	60.57	(2, 35)	60.57	0.00
		0.25	(1, 34, 0, 0)	59.89	(1, 34)	59.89	0.00
		0.1	(0, 33, 0, 0)	58.67	(0, 33)	58.67	0.00
		0.05	(0, 32, 0, 0)	57.95	(0, 32)	57.95	0.00
		0.01	(0, 32, 0, 0)	57.30	(0, 32)	57.30	0.00
	0.1	1	(1, 34, 0, 0)	59.10	(1, 34)	59.10	0.00
		0.8	(1, 33, 0, 0)	58.99	(1, 33)	58.99	0.00
		0.5	(1, 33, 0, 0)	58.78	(1, 33)	58.78	0.00
		0.25	(0, 33, 0, 0)	58.49	(0, 33)	58.49	0.00
		0.1	(0, 32, 0, 0)	57.75	(0, 32)	57.75	0.00
		0.05	(0, 32, 0, 0)	57.45	(0, 32)	57.45	0.00
		0.01	(0, 32, 0, 0)	57.19	(0, 32)	57.19	0.00

**Table 9:** Comparison of the (*s*, *S*) policy and $(s_1,S_1,s_2,S_2)$ policy with constant lead time $L_o=1$ when $K_o=10$ and $b=100$ ($\lambda =5$, $h=1$, $c_o=c_e=5$)

$K_e/K_o$	$1/\nu$	$\mu /\nu$	$(s_1^*,S_1^*,s_2^*,S_2^*)$	$\mathbb {E}[C_1]$	$(s^*,S^*)$	$\mathbb {E}[C_2]$	%Gap$_{12}$
3	10	1	(10, 21, 88, 101)	107.79	(73, 103)	135.05	20.19
		0.8	(10, 21, 76, 104)	102.49	(67, 94)	133.28	23.10
		0.5	(10, 20, 80, 103)	90.43	(55, 84)	127.38	29.01
		0.25	(10, 21, 84, 94)	68.56	(22, 58)	102.56	33.15
		0.1	(10, 21, 92, 93)	54.20	(11, 28)	75.17	27.89
		0.05	(10, 21, 89, 97)	48.49	(10, 23)	58.10	16.53
		0.01	(10, 21, 83, 84)	42.36	(10, 21)	44.50	4.81
	1	1	(19, 34, 0, 0)	53.76	(19, 34)	53.76	0.00
		0.8	(18, 35, 0, 0)	53.51	(18, 35)	53.51	0.00
		0.5	(17, 32, 0, 0)	52.79	(17, 32)	52.79	0.00
		0.25	(15, 29, 0, 0)	50.32	(15, 29)	50.32	0.00
		0.1	(11, 25, 0, 0)	46.94	(11, 25)	46.94	0.00
		0.05	(10, 23, 16, 26)	43.33	(11, 23)	44.58	2.79
		0.01	(10, 21, 17, 25)	41.82	(10, 21)	42.07	0.59
	0.5	1	(14, 28, 0, 0)	46.53	(14, 28)	46.53	0.00
		0.8	(14, 26, 0, 0)	46.18	(14, 26)	46.18	0.00
		0.5	(13, 25, 0, 0)	45.73	(13, 25)	45.73	0.00
		0.25	(12, 25, 0, 0)	44.78	(12, 25)	44.78	0.00
		0.1	(11, 23, 0, 0)	43.34	(11, 23)	43.34	0.00
		0.05	(10, 22, 0, 0)	42.43	(10, 22)	42.43	0.00
		0.01	(10, 21, 0, 0)	41.59	(10, 21)	41.59	0.00
	0.25	1	(11, 23, 0, 0)	43.06	(11, 23)	43.06	0.00
		0.8	(11, 24, 0, 0)	43.11	(11, 24)	43.11	0.00
		0.5	(11, 22, 0, 0)	42.67	(11, 22)	42.67	0.00
		0.25	(11, 22, 0, 0)	42.34	(11, 22)	42.34	0.00
		0.1	(10, 21, 0, 0)	41.85	(10, 21)	41.85	0.00
		0.05	(10, 21, 0, 0)	41.69	(10, 21)	41.69	0.00
		0.01	(10, 21, 0, 0)	41.51	(10, 21)	41.51	0.00
	0.1	1	(10, 22, 0, 0)	41.89	(10, 22)	41.89	0.00
		0.8	(10, 21, 0, 0)	41.77	(10, 21)	41.77	0.00
		0.5	(10, 21, 0, 0)	41.69	(10, 21)	41.69	0.00
		0.25	(10, 21, 0, 0)	41.57	(10, 21)	41.57	0.00
		0.1	(10, 21, 0, 0)	41.55	(10, 21)	41.55	0.00
		0.05	(10, 21, 0, 0)	41.44	(10, 21)	41.44	0.00
		0.01	(10, 21, 0, 0)	41.43	(10, 21)	41.43	0.00

We start by redefining the $(s_1,S_1,s_2,S_2)$ policy. Here, the retailer needs to use the inventory position (i.e., inventory level plus outstanding order) instead of the inventory level for taking order decisions. Therefore, the policy can be summarized as follows: (1) when the inventory position is less than or equal to $s_1$ and the supplier is *on*, a regular order is placed to increase the inventory position up to $S_1$; (2) when the inventory position is less than or equal to $s_2$ upon supplier’s switch to the *off* mode, an emergency order is triggered to raise the inventory position to $S_2$. An emergency order is given based on the inventory position as it is not generally economically viable to place an emergency order if there is any outstanding order in the system.

Table 9 displays the results for the base parameter set with $L_o=1$, $b=100$ and $K_o=10$. The optimal policy parameters show similar behavior with respect to the changes in the problem parameters as in the zero lead time case. Here, we also observe exercising $(s_1,S_1,s_2,S_2)$ policy yields considerable savings when the expected duration of *off* period is long.

The data instances with $1/\nu \le 1$ correspond to environments where the expected length of the *off* period is shorter than or equal to the lead time. The retailer is always prepared to respond to the demand during lead time. Thus, she is less vulnerable to disruptions that are potentially shorter than the lead time. This explains why emergency orders are not used for those instances.

### Numerical results of exponentially distributed regular replenishment lead time case

In this subsection, we present some numerical examples for the model with exponentially distributed regular lead time. We compute the optimal decisions and associated costs for $(s_1,S_1,s_2,S_2)$ and (*s*, *S*) policies using the models in Sect. 4 and "Appendix 4." Table 10 reports the results for the base parameter set with $L_o=1$, $b=100$, and $K_o=10$.

The results imply mostly similar insights with the other lead time scenarios. The expected duration of the *off* period and supplier’s availability strongly affect the performance of $(s_1,S_1,s_2,S_2)$ policy. The disruption orders are especially valuable when the supplier’s availability is low. As the supplier’s availability decreases ($\mu /\nu$ increases) for a fixed $1/\nu$, we observe higher discrepancy between $s_1$ and *s*, and $S_1$ and *S*. Considering the lead time, (*s*, *S*) policy inflates the stock levels when the expected length of *on* period is relatively short compared to the *off* period.

**Table 10:** Comparison of the (*s*, *S*) policy and $(s_1,S_1,s_2,S_2)$ policy with exponential lead time $\ell =1$ when $K_o=10$ and $b=100$ ($\lambda =5$, $h=1$, $c_o=c_e=5$)

$K_e/K_o$	$1/\nu$	$\mu /\nu$	$(s_1^*,S_1^*,s_2^*,S_2^*)$	$\mathbb {E}[C_1]$	$(s^*,S^*)$	$\mathbb {E}[C_2]$	%Gap$_{12}$
3	10	1	(11, 65, 101, 114)	124.51	(74, 124)	146.43	14.97
		0.8	(12, 63, 99, 112)	118.65	(69, 114)	141.74	16.29
		0.5	(14, 56, 96, 109)	105.79	(56, 94)	130.59	18.99
		0.25	(15, 48, 92, 104)	88.06	(33, 64)	110.21	20.10
		0.1	(16, 41, 89, 101)	71.28	(21, 42)	82.90	14.02
		0.05	(17, 38, 88, 100)	63.87	(19, 38)	69.98	8.74
		0.01	(18, 35, 87, 99)	56.95	(18, 35)	58.19	2.12
	1	1	(0, 41, 25, 41)	66.73	(36, 65)	78.71	15.22
		0.8	(0, 42, 26, 42)	67.68	(33, 61)	75.42	10.27
		0.5	(29, 53, 0, 0)	69.87	(29, 53)	69.87	0.00
		0.25	(23, 39, 25, 45)	64.14	(24, 45)	64.17	0.05
		0.1	(20, 36, 23, 39)	59.44	(21, 40)	59.53	0.15
		0.05	(19, 34, 22, 37)	57.42	(19, 37)	57.50	0.12
		0.01	(18, 33, 21, 35)	55.55	(18, 35)	55.57	0.04
	0.5	1	(0, 33, 16, 33)	58.34	(33, 60)	74.38	21.57
		0.8	(0, 34, 17, 34)	59.15	(30, 56)	71.21	16.94
		0.5	(0, 37, 20, 37)	62.03	(26, 49)	66.07	6.10
		0.25	(22, 42, 0, 0)	61.20	(22, 42)	61.20	0.00
		0.1	(20, 38, 0, 0)	57.75	(20, 38)	57.75	0.00
		0.05	(19, 36, 0, 0)	56.46	(19, 36)	56.46	0.00
		0.01	(18, 35, 0, 0)	55.33	(18, 35)	55.33	0.00
	0.25	1	(0, 28, 10, 28)	53.04	(31, 58)	72.36	26.71
		0.8	(0, 28, 11, 28)	53.58	(29, 54)	69.30	22.68
		0.5	(0, 30, 13, 30)	55.60	(25, 47)	64.43	13.71
		0.25	(22, 41, 0, 0)	60.02	(22, 41)	60.02	0.00
		0.1	(19, 37, 0, 0)	57.13	(19, 37)	57.13	0.00
		0.05	(19, 36, 0, 0)	56.11	(19, 36)	56.11	0.00
		0.01	(18, 35, 0, 0)	55.25	(18, 35)	55.25	0.00
	0.1	1	(0, 24, 6, 24)	49.07	(30, 56)	71.23	31.10
		0.8	(0, 24, 6, 24)	49.39	(28, 52)	68.24	27.63
		0.5	(0, 25, 7, 25)	50.45	(24, 46)	63.57	20.64
		0.25	(0, 28, 10, 28)	53.29	(21, 40)	59.44	10.35
		0.1	(19, 37, 0, 0)	56.84	(19, 37)	56.84	0.00
		0.05	(18, 36, 0, 0)	55.96	(18, 36)	55.96	0.00
		0.01	(18, 35, 0, 0)	55.22	(18, 35)	55.22	0.00

## Concluding remarks

Disruption information can be seen as an asset for inventory systems subject to supply disruptions. The information about the likelihood of disruptions can provide means to develop a mitigation strategy against lost sales risk. In this study, we have considered an emergency ordering opportunity just before or when disruption occurs as a strategy to decrease shortage risk during the disrupted period. According to this strategy, the replenishment orders are placed following a combination of two order-up-to policies, $(s_1,S_1,s_2,S_2)$, one for the regular mode and the other for the disrupted mode. In order to analyze the benefits of this policy, we have studied a continuous-review stochastic inventory system with an unreliable supplier. We consider Poisson demand, lost sales, and different lead time scenarios. Assuming that supplier’s availability randomly alternates among *on* and *off* modes, we describe the system by CTMC models for zero and exponentially distributed regular lead time cases. CTMC models are used to derive the stationary distribution of inventory level and the resulting distributions are utilized to formulate the exact expressions of the expected total cost in the $(s_1,S_1,s_2,S_2)$ policy. We rely on an enumeration search to find the optimal policy parameters. The properties drawn from the CTMC enable us to reduce the computational effort.

We attempt to show benefits of $(s_1,S_1,s_2,S_2)$ policy by conducting a numerical study based on the parameter sets in Berk and Arreola-Risa (1994) and Moinzadeh and Aggarwal (1997). We compare the results of $(s_1,S_1,s_2,S_2)$ policy with the inventory model without opportunity for disruption orders (i.e., the (*s*, *S*) policy). In addition to zero and exponentially distributed lead time scenarios, we also numerically investigate the nonzero constant lead time case. Similar results and insights generally hold for all lead time scenarios. The $(s_1,S_1,s_2,S_2)$ policy yields remarkable cost savings in case of high unit lost sales cost, high likelihood of long disrupted periods, and low percentage of supplier’s availability. One can consider unit lost sales cost high, if it gets a large value compared to the other cost parameters. However, the policy is less valuable for systems with a high fixed cost of ordering. As it becomes more expensive to give an emergency order, it converges to the (*s*, *S*) policy. Note that the savings would be even greater if the parameters of (*s*, *S*) policy are set without considering the supply disruption.

Long disrupted periods might be faced in cases such as pandemic, earthquake, labor strike, and material shortage. The model has a considerable potential of applicability in such cases if the supplier would agree to give priority for transferring available inventory to the retailer at the disruption time. We believe our results have implications in a variety of sectors such as original equipment manufacturers, gas and oil industries as well as wind power generation, where the downtime is expensive and the failure time might be lengthy.

## Figures and Tables

**Fig. 3:**
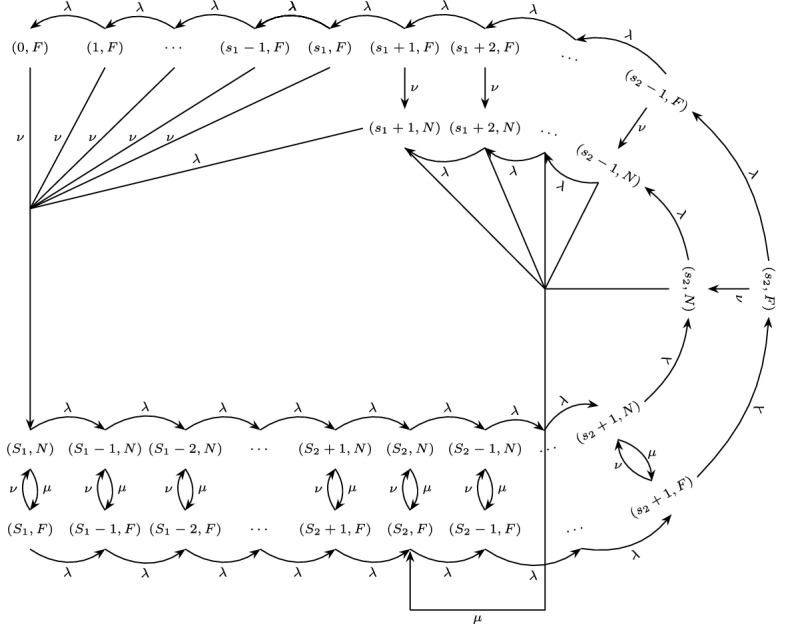
Markov chain transition diagram for the $(s_1,S_1,s_2,S_2)$ policy

**Fig. 4:**
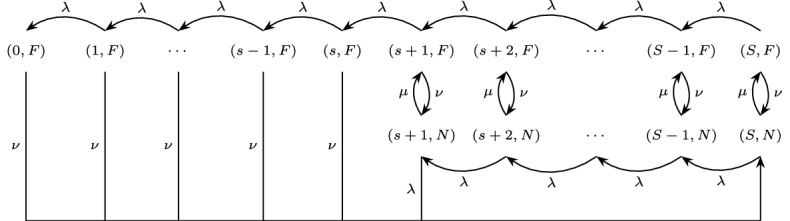
Markov chain transition diagram for the (*s*, *S*) policy with zero lead time

**Fig. 6:**
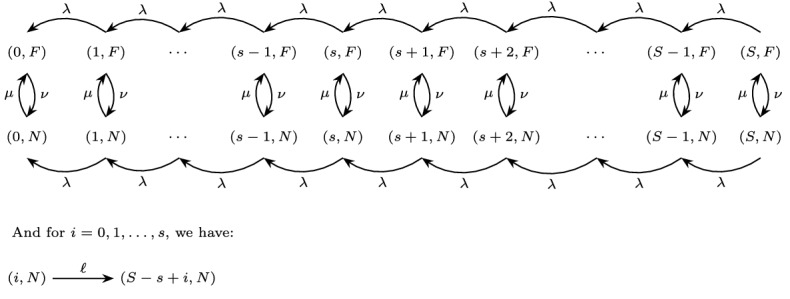
Markov chain transition diagram for the (*s*, *S*) policy with exponential lead time

## Appendix 1: Transition diagram for $\varvec{(s_1,S_1,s_2,S_2)}$ policy with zero lead time

See Fig. 3.

## Appendix 2: CTMC model of $\varvec{(s,S)}$ policy with zero lead time

The transition diagram of  the (*s*, *S*) policy is shown  in Fig. 4.

Balance equations in the (*s*, *S*) policy, which are constructed by equating total flow rate into any state with the total flow rate out of that same state, are as follows:

27 $$\begin{aligned}&\nu \mathcal {P}_{(0,F)} = \lambda \mathcal {P}_{(1,F)}. \end{aligned}$$

28 $$\begin{aligned}&(\nu +\lambda ) \mathcal {P}_{(i,F)} = \lambda \mathcal {P}_{(i+1,F)}, \ \forall \ i=1,\ldots ,s_. \end{aligned}$$

29 $$\begin{aligned}&(\nu +\lambda ) \mathcal {P}_{(i,F)} = \lambda \mathcal {P}_{(i+1,F)} + \mu \mathcal {P}_{(i,N)}, \ \forall \ i=s+1,\ldots ,S-1. \end{aligned}$$

30 $$\begin{aligned}&(\nu +\lambda ) \mathcal {P}_{(S,F)} = \mu \mathcal {P}_{(S,N)}. \end{aligned}$$

31 $$\begin{aligned}&(\mu +\lambda ) \mathcal {P}_{(i,N)} = \lambda \mathcal {P}_{(i+1,N)} + \nu \mathcal {P}_{(i,F)}, \ \forall \ i=s+1,\ldots ,S-1. \end{aligned}$$

32 $$\begin{aligned}&(\mu +\lambda ) \mathcal {P}_{(S,N)} = \lambda \mathcal {P}_{(s+1,N)} + \nu \Big (\sum _{i=0}^{s}\mathcal {P}_{(i,F)}+\mathcal {P}_{(S,F)}\Big ). \end{aligned}$$

In what follows, we describe how to derive the steady-state probabilities. For ease of exposition, let us denote the following notation:

$$\begin{aligned} c_1 = \frac{\mu }{\nu +\lambda }, \ c_2 = \frac{\lambda }{\mu +\lambda }, \ c_3 = \frac{\lambda }{\nu +\lambda },~ \ \text{ and }~ \ c_4 = \frac{\nu }{\mu +\lambda }. \end{aligned}$$

*Step 1.* Considering two sets of Equations (29) and (31) together, for $i=s+1,\ldots ,S-1$, we have:

33 $$\begin{aligned} \begin{aligned} \mathcal {P}_{(i,N)} = \xi _1^i \mathcal {P}_{(S,F)} \ and \ \mathcal {P}_{(i,F)} = \xi _2^i \mathcal {P}_{(S,F)}, \end{aligned} \end{aligned}$$

where

$$\begin{aligned} \begin{aligned} \xi _1^i = \frac{c_2 \xi _1^{i+1} + c_3 c_4 \xi _{2}^{i+1}}{1-c_1c_4}, \ \xi _2^i = c_3 \xi _2^{i+1} + \frac{c_1c_2 \xi _1^{i+1} + c_1 c_3c_4 \xi _{2}^{i+1}}{1-c_1c_4}, \ \forall \ i=s+1,\ldots ,S-1, \end{aligned} \end{aligned}$$

and $$\xi _1^{S} = 1/c_1$$, $$\xi _2^{S}=1$$.

*Step 2.* Using Equation (33), we can obtain $\mathcal {P}_{(s_1+1,N)}$ w.r.t $\mathcal {P}_{(S,F)}$ and substitute in Equation (32). Then, solving Equation (32) one can find $\mathcal {P}_{(S,F)}$ w.r.t $\mathcal {P}_{(0,F)}$. Next, using the set of Equations in (33) and (28) we can derive all steady-state probabilities w.r.t $\mathcal {P}_{(0,F)}$. Finally, summing up all of them we can find the steady-state probabilities.

By doing two-step algorithm above, the steady-state probabilities are obtained as follows:

34 $$\begin{aligned} \begin{aligned}&\mathcal {P}_{(S,F)} = \theta \mathcal {P}_{(0,F)}, \\ {}&\mathcal {P}_{(S,N)} = \frac{\theta }{c_1} \mathcal {P}_{(0,F)}, \\ {}&\mathcal {P}_{(i,N)} = \xi _1^i \mathcal {P}_{(0,F)}, \ \forall \ i=s+1,\ldots ,S-1, \\ {}&\mathcal {P}_{(i,F)} = \xi _2^i \mathcal {P}_{(0,F)}, \ \forall \ i=s+1,\ldots ,S-1,\\ {}&\mathcal {P}_{(i,F)} = (\frac{1}{c_1})^{i-1} \mathcal {P}_{(0,F)}, \ \forall \ i=1,\ldots ,s, \end{aligned} \end{aligned}$$

where

$$\begin{aligned} \mathcal {P}_{(0,F)}= & {} \Big (1+ \frac{\nu }{\lambda } \sum _{i=1}^{s} (\frac{1}{c_1})^{i-1} + (1+\frac{S(S-1)-s(s+1)}{2}) (1+\frac{1}{c_1}) \theta \Big )^{-1},\\ \theta= & {} \frac{\nu \big (1+ \frac{\nu }{\lambda } \sum _{i=0}^{s-1}(\frac{\nu +\lambda }{\lambda })^i \big )}{(\mu +\lambda )(\frac{\nu +\lambda }{\mu }) -\lambda \xi _1^{s+1} -\nu }. \end{aligned}$$

## Appendix 3: Transition diagram for $\varvec{(s_1,S_1,s_2,S_2)}$ policy with exponential lead time

See Fig. 5.

## Appendix 4: CTMC model of $\varvec{(s,S)}$ policy with exponential lead time

The transition diagram of  the (*s*, *S*) policy is presented  in Fig. 6.

## Appendix 5: Proofs

**Proof of Theorem** 1. Consider the corresponding embedded Markov chain (embedded M.C) of our CTMC. We can easily verify that the embedded M.C is irreducible as all states are in one class and also aperiodic as one cannot return to the same state after a specific number of steps. Aperiodicity is a class property, which implies that if one of the states is not aperiodic, the other states in the same class are not also aperiodic. Consider state $(S_1,F)$, this state can be visited in any number of steps. Hence, the embedded M.C is aperiodic. In a Markov chain with a finite number of states, all states cannot be transient; therefore, the given Markov chain is recurrent. Moreover, in a Markov chain with a finite number of states, all recurrent states are positive recurrent. An irreducible, aperiodic, and positive recurrent Markov chain is ergodic, and the proof follows.

**Proof of Proposition** 1. Starting from Equation (15), we can find $\pi _{S_2} = \mathcal {P}_{(S_2,F)}+\mathcal {P}_{(S_2,N)}$ w.r.t $\mathcal {P}_{(S_1,F)}$, $\mathcal {P}_{(s_1+1,N)}$, and $\mathcal {P}_{(0,F)}$. Using Equations (13) and (14), we can find $\pi _{S_2-1}$ w.r.t $\mathcal {P}_{(S_1,F)}$, $\mathcal {P}_{(s_1+1,N)}$, and $\mathcal {P}_{(0,F)}$ as well. Comparing the coefficients of $\mathcal {P}_{(S_1,F)}$, $\mathcal {P}_{(s_1+1,N)}$ and $\mathcal {P}_{(0,F)}$ in resulting expressions for $\pi _{S_2}$ and $\pi _{S_2-1}$, we observed that they are equal to each other. Repeating this procedure we found that the coefficients of $\mathcal {P}_{(S_1,F)}$, $\mathcal {P}_{(s_1+1,N)}$, and $\mathcal {P}_{(0,F)}$ in all $\pi _{i}$ for $i=s_2+1,\ldots ,S_2$ are the same. It implies the result.

**Proof of Proposition** 2. Starting from $\pi _{S_1}$, we have $\pi _{S_1} = \mathcal {P}_{(S_1,F)}+\mathcal {P}_{(S_1,N)} = (1+\frac{1}{c_1})\mathcal {P}_{(S_1,F)}$. Considering Equation (11), $\pi _{i} = \xi _1^i \mathcal {P}_{(S_1,F)} + \xi _2^i \mathcal {P}_{(S_1,F)} = (\xi _1^i+\xi _2^i) \mathcal {P}_{(S_1,F)}$, for $i=S_2+1,\ldots ,S_1$. One can easily verify that $\xi _1^i+\xi _2^i=1+\frac{1}{c_1}$ which yields the result.

## Appendix 6: Validation of the analytical results by simulation

We conduct a simulation study to validate our analytical results presented in Sects. 3.2 and 3.3. We calculate the expected cost rate for some randomly selected instances with varying inventory policy parameters through simulation and Equation (21) and compare the results. We use Arena 14.5 student version for conducting the simulation. The numbers given in Table 11 for the simulation method are results of 10 replications each with a run length of one million days and warm-up period of 1000 days. We use the base parameter set $\lambda =5$, $h=1$, $b=10$, $c_o=5$, $c_e=5$, $K_o=10$, $K_e=30$, $\nu =1$, and $\mu =1$, while varying the inventory policy parameters. Table 11 shows the expected cost calculated by Equation (21) and simulation and also the percentage deviation between analytical and simulation results. Our numerical analysis reveals the accuracy of our analytical results.

**Table 11:** Comparison of analytical and simulation results

$(s_1, S_1, s_2, S_2)$	Analytical results	Simulation	Gap
(2, 12, 5, 15)	41.13	41.13	0.00
(8, 15, 10, 12)	45.48	45.48	0.00
(0, 10, 3, 7)	40.95	40.95	0.00
(10, 23, 12, 27)	46.29	46.29	0.00
(2, 11, 4, 8)	40.85	40.85	0.00
(9, 20, 10, 13)	43.90	43.90	0.00
(0, 9, 4, 13)	41.46	41.45	0.01
(9, 19, 11, 15)	45.11	45.11	0.00
(5, 16, 7, 22)	42.08	42.09	− 0.01
(7, 22, 14, 20)	48.66	48.66	0.00

We also utilize a simulation-optimization framework based on a complete enumeration approach to validate the accuracy of the optimal policy parameters found by the enumeration algorithm given in Sect. 3.4. We do this validation process for all of the numerical results reported in Sect. 5.
